# Echo Chains as a Linear Mechanism: Norm Inflation, Modified Exponents and Asymptotics

**DOI:** 10.1007/s00205-021-01697-6

**Published:** 2021-07-30

**Authors:** Yu Deng, Christian Zillinger

**Affiliations:** 1 Department of Mathematics, University of Southern California, Los Angeles, CA 90089 USA; 2 BCAM – Basque Center for Applied Mathematics, Mazarredo 14, E48009 Bilbao, Basque Country Spain; 3 Present Address: Karlsruhe Institute of Technology, Englerstraße 2, 76131 Karlsruhe, Germany

## Abstract

In this article we show that the Euler equations, when linearized around a low frequency perturbation to Couette flow, exhibit norm inflation in Gevrey-type spaces as time tends to infinity. Thus, echo chains are shown to be a (secondary) linear instability mechanism. Furthermore, we develop a more precise analysis of cancellations in the resonance mechanism, which yields a modified exponent in the high frequency regime. This allows us, in addition, to remove a logarithmic constraint on the perturbations present in prior works by Bedrossian, Deng and Masmoudi, and to construct solutions which are initially in a Gevrey class for which the velocity asymptotically converges in Sobolev regularity but diverges in Gevrey regularity.

## Introduction

In this article our aim is to develop a further understanding of the long-time asymptotic behavior of the 2D Euler equations near Couette flow

1 $$\begin{aligned} \partial _t \omega + y \partial _x \omega + v \cdot \nabla \omega =0 \end{aligned}$$

in an infinite channel ${\mathbb {T}}\times {\mathbb {R}}$. As the velocity field is incompressible, all $L^p$ norms of the vorticity $\omega$ are conserved and the equation is nonlinearly stable in this sense. Furthermore, the linearized problem exhibits weak convergence of the vorticity in $L^2$ and as a consequence in the linear problem $v - \langle v \rangle _x$ converges strongly in $L^2$ as $t \rightarrow \infty$. This behavior is known as linear inviscid damping in analogy to the similar phenomenon of Landau damping in plasma physics. While the linearized problem possesses an explicit solution and exhibits linear inviscid damping for any initial data in $H^s, s\geqq 0$, the question of stability and asymptotic behavior of more general shear flows or the nonlinear problem have been a very active area of research in recent years. In particular, we mention the following publications:

- In [3] Bedrossian and Masmoudi established nonlinear inviscid damping for Gevrey 2 regular perturbations around Couette flow in an infinite periodic channel. Their method of proof has further been extended to the setting of Landau damping [4, 9].
- These results were recently extended to the case of compactly supported Gevrey regular perturbations to Taylor–Couette flow in [6].
- In [5] the first author and Masmoudi constructed solutions of the Euler equations, such that the initial data is Gevrey 2 close to Couette flow, the solution is well-understood for a finite time and within that finite time the solution exhibits growth along echo chains consistent with a loss of Gevrey 2 regularity. Here, we in particular stress that due to the challenging control of nonlinear corrections, these solutions *do not* capture the full echo chains (the finite time includes about half of a chain) and hence *do not* rule out subsequent asymptotic stability. Furthermore, that work imposes a logarithmic smallness restriction (see Section 4 for a further discussion). Removing this restriction is a key challenge in establishing our modified scattering in Section 5.3.
- Concerning the problem of linear inviscid damping, the case of a finite-periodic channel was shown to behave qualitatively differently in a work by the second author [14] in that stability results are limited to (sharp) $H^{3/2-}$ or weighted $H^2$, [12], Sobolev regularity of the vorticity. While sufficient to establish linear inviscid damping with the optimal decay rates of the velocity perturbation, this leaves a large gap compared to the Gevrey regularity requirement of existing nonlinear results. Indeed, in [7, 8] Ionescu and Jia further impose a compact support assumption to establish linear stability in Gevrey spaces.
- This gap, together with the physical observation of damping, suggests that damping of the velocity field might be a more robust mechanism than the stability of the vorticity. A first modest step in this direction can be found in [16] where the second author considered special types of forcing of the 2D Euler and Navier-Stokes equations and showed that damping may persist despite instability of the vorticity.

In this article, we stress the point of view that for the question of nonlinear inviscid damping in addition to the question of linear stability of shear flows in Sobolev and Gevrey regularity, one should consider the following two observations:

1. Existing works study the infinite time asymptotic stability of the vorticity in higher Sobolev regularity or Gevrey regularity, from which (linear) inviscid damping then follows as a corollary. However, this is a strictly stronger condition than the physically observed phenomenon of inviscid damping, which is the convergence of the velocity field. Indeed, we show that the linearized problem exhibits solutions which exhibit norm inflation due to complete echo chains. Here, the velocity asymptotically converges despite the divergence of the vorticity as time tends to infinity.
2. In the study of nonlinear inviscid damping, the main challenge is given by cascades of resonances, also known as echo chains. This mechanism is, however, not present in the linearized problem around shear flows due to the decoupling structure in Fourier space of these equations. In this article, we identify echo chains as a *secondary linear* mechanism, where we linearize around an arbitrarily small low-frequency perturbation around Couette flow. More precisely, we show that in arbitrarily small neighborhoods of Couette flow (with respect to local norms) there are *traveling wave-like* solutions of the form
  $$\begin{aligned} \omega (t,x,y)&=-1 + c \cos (x-ty), \\ v(t,x,y)&= (y,0) - \nabla ^{\perp } \frac{1}{1+t^2} c \cos (x-ty), \end{aligned}$$
  and study (a simplification of) the linearized problem around this wave. For simplicity of calculations and presentation, in this article we consider a single mode perturbation by $c\cos (x)$ with *c* small. However, we think that an extension to more general low-frequency perturbations, while technically tedious, should follow by similar arguments. We show that this (simplified) linearized problem exhibits the same echo chains and norm-inflation results as for the nonlinear problem, globally in time. Thus, *echo chains are a linear mechanism*. Furthermore, we identify a critical regularity threshold at which stability of the vorticity in Gevrey regularity fails, but where damping of the velocity nevertheless persists.

In order to introduce our model consider the Euler equations near Couette flow (1) and change to Lagrangian coordinates (with respect to Couette flow) $(x+ty,y)$. Then our equation is given by

$$\begin{aligned} \partial _t\omega + \nabla ^\perp \varDelta _{t}^{-1} \omega \cdot \nabla \omega =0, \end{aligned}$$

where $\varDelta _t=\partial _x^2+(\partial _y-t\partial _x)^2$ and $\omega$ denotes the perturbation. Let now $P_N$ denote a Littlewood–Payley projection to a large dyadic scale *N*. Then the projection of the equation yields

2 $$\begin{aligned} \partial _tP_N \omega + P_N (\nabla ^\perp \varDelta _t^{-1}\omega \cdot \nabla \omega )=0. \end{aligned}$$

We may then further decompose the nonlinearity by frequency-localizing each factor:

3 $$\begin{aligned} \sum _{N_1+N_2 \approx N} P_N (\nabla ^\perp \varDelta _t^{-1}P_{N_1}\omega \cdot \nabla P_{N_2}\omega ). \end{aligned}$$

Here, we obtain several regimes. If $N_1 \approx N_2$, we may freely integrate by parts and trade the inverse Laplacian $\varDelta _t^{-1}$ for time decay. Similarly, if $N_2\approx N, N_1\ll N_2$, we may use the incompressibility (to obtain cancellations at leading order) and trade two derivatives for decay of $\varDelta _t^{-1}$ to obtain a time-decreasing energy estimate.

The main source of possible growth and instability in the nonlinear problem is thus given by the interaction of the *low-frequency part of the vorticity*, $N_2 \ll N$, and the *high-frequency part of the velocity*, $N_1 \approx N$. In our model we now *fix the low frequency part* $\omega _{\text {low}}=c\cos (x)$ with *c* small and consider the (simplified) linearized problem for a perturbation, which, with slight abuse of notation, we again denote by $\omega$:

4 $$\begin{aligned} \partial _t\omega + c \sin (x) \partial _y \varDelta _{t}^{-1} \omega =0. \end{aligned}$$

We stress that $\omega _{\text {low}}$ is an exact solution of the nonlinear Euler equations (see Lemma 1), which we call a *traveling wave-like solution*. We remark that $\omega _{low}$ is not stationary in Eulerian coordinates, but is stationary in coordinates $(x+ty,y)$ moving with Couette flow (we hence view it as a wave moving with the flow). Our model thus corresponds to a secondary linearization around $v=(y,0)+ c \nabla ^{\perp }\frac{1}{1+t^2}\cos (x-ty)$, where we neglected the transport by $\frac{c\sin (x)}{1+t^2}\partial _y$ for simplicity.

Our main result is then that this model exhibits the same *echo chains* as the full nonlinear Euler equations near Couette flow (in Gevrey regularity, see [2, 5]), but further exhibits *modified scattering* and linear *inviscid damping* in the sense the velocity perturbation strongly converges as $t\rightarrow \infty$, but that the vorticity diverges in $H^{s}$ for any $s>-1$. The secondary linearization captures the nonlinear resonance mechanism and, for a critical class of data, exhibits damping and blow-up at the same time.

### Theorem 1

(Summary) Let $0<c<0.2$, then there exists $C>0$ such that for any $s\in {\mathbb {R}}$ there exists solutions of (4) with $\omega _0 \in {\mathcal {G}}_{C,\frac{1}{2}}$ (Gevrey class, see Section 1.1.1) such that $\omega (t)$ converges to a limit $\omega _{\infty }$ in $H^{\sigma }, \sigma \leqq s$, but diverges in $H^{\sigma }, \sigma >s$. In particular, in the case $s\geqq 0$ we note that *damping*, that is asymptotic convergence of the velocity field, holds, while *asymptotic stability* in Gevrey regularity fails.

Furthermore, the constant *C* is (almost) optimal in the sense that there exists a (larger) constant $C_0>0$ such that for any $C_1>2 C_0$ if $\omega _0 \in {\mathcal {G}}_{C_1,\frac{1}{2}}$, then $\omega (t)\in {\mathcal {G}}_{C_1-C_0, \frac{1}{2}}$ for all times and *u*(*t*) converges in ${\mathcal {G}}_{C_1-C_0, \frac{1}{2}}$.

As we discuss in the ensuing subsection, a key challenge in this result is that the (nonlinear) growth mechanism of echo chains stops after a finite time for any given chain. Hence, in order to obtain non-trivial asymptotic behavior like blow-up it is necessary to work with countably many echo chains of increasing length. In particular, one has to allow for arbitrarily long chains, which had been ruled out by logarithmic smallness constraints in prior works. A key effort of this article in Sections 4 and 5 is then to remove this constraint and show that solutions behave qualitatively differently in that high frequency regime than in the previously considered low frequency case.

### Outline and Challenges

In their seminal work Bedrossian and Masmoudi [3] established nonlinear asymptotic stability of Couette flow under sufficiently small Gevrey 2 regular perturbations. Furthermore, they showed that the perturbation of the velocity field decays (at algebraic rates) as time tends to infinity – a phenomenon which is known as *inviscid damping* in analogy to Landau damping in plasma physics. Following this result, there has been much renewed interest in inviscid damping, both linear and nonlinear, also around other flows. In particular, it turns out that the linearized problem (also around more general shear flows) is stable already in relatively low Sobolev regularity and that, unless one assumes compact support of perturbations, geometries with boundary do not allow for (linear) stability in high Sobolev regularity.

The reason for this large gap in regularity requirements – Gevrey as opposed to Sobolev – is tied to a physical phenomenon called *fluid echoes* [13]. As we recall in Section 3 here two perturbations at frequencies *k* and *l* in *x* (and suitable frequencies in *y*) may interact by means of the nonlinearity and introduce an (at first quadratically small) correction at frequency $k+l$. This perturbation may then become resonant at a later time and may thus yield a large contribution to the velocity field. We say that the perturbations at frequency *k* and *l* result in an *echo* at frequency $k+l$ at that time. In [3] it is then estimated by means of a toy model that a chain of such echoes, where frequency *k* causes an echo at frequency $k-1$, which in turn causes an echo at frequency $k-2$, $\ldots$ could lead to Gevrey 2 norm inflation. Indeed in [5] the first author and Masmoudi showed that such a chain may indeed form in the nonlinear equations (at least from *k* to $\frac{k}{2}$).

Since the linearized equations around a shear flow (which is independent of *x*) decouple with respect to frequency in *x*, these equations do not include the echo mechanism and hence echo chains are commonly understood to be a nonlinear effect. In this article we argue that it might be useful to instead view them as a *secondary linear effect*, where one should linearize around **traveling wave-like** solutions

$$\begin{aligned} \omega (t,x,y)= -1 + c \cos (x-ty) \end{aligned}$$

instead of around a shear flow. We observe that in coordinates $(x-ty,y)$ moving with Couette flow such a wave is stationary and concentrated on two modes $+1, -1$ (in *x* and 0 in *y*). The heuristic of our echo chain construction is then the following:

- We study the (simplified) linear problem around this wave, where we initially introduce a perturbation at frequency $(k_0, \eta )$.
- These equations include the echo mechanism in the sense that the *underlying wave* (at frequencies $(\pm 1, 1)$) interacts with the *perturbation* at frequency $(k_0,\eta )$, which results in *echoes* at frequencies $(k_0+1, \eta )$ and $(k_0-1, \eta )$ at around a resonant time $t \approx \frac{\eta }{k_0}$.
- In turn the contribution at frequency $(k_0-1, \eta )$ by the echo can be viewed as new perturbation at a new initial time.
- The interaction of this perturbation at frequency $(k_0-1,\eta )$ with the underlying wave results in echoes at frequencies $(k_0-2, \eta )$ and $(k_0, \eta )$ at around the resonant time $\frac{\eta }{k_0-1}$.
- We may iterate along this chain until we reach the last resonance at time $\frac{\eta }{1}$, which corresponds to the largest norm inflation.
- Furthermore, in Theorem 9 we combine infinitely many such chains to show that this inflation is sharp in the sense that there exist Gevrey 2 regular initial data which exhibit infinitely many echo chains and asymptotically blow up in $H^s$, but still converge in $H^{s-\varepsilon }$. *Damping of the velocity field persists despite blow-up of the vorticity*. When requiring higher Gevrey 2 regularity, that is with a larger constant, this growth is hidden by the rapid frequency decay and the solution remains Gevrey regular for all times and in the limit $t \rightarrow \infty$.

In order to make this heuristic rigorous we first, equivalently, formulate our equation as an infinite ODE system (7) in Section 2, which by the structure of the underlying wave includes only nearest neighbor interaction and is of the form

$$\begin{aligned} \partial _t\omega (t,l,\eta ) + a(t,l+1,\eta ) \omega (t,l+1,\eta ) - a(t,l-1,\eta ) \omega (t,l-1, \eta )=0 \end{aligned}$$

for all *l* (the equation decouples with respect to $\eta$). The main aim of this article, which culminates in Theorem  8 of Section 5.2, is to show that on any time interval *I* centered around the resonant time $\frac{\eta }{k}$ (see Section 5) *the evolution is largely determined by just the* ${\varvec{three \,modes}}$ $k+1, k, k-1$. As an important preliminary step, we hence study the associated homogeneous problem for these three modes (that is, neglecting all other modes in the evolution) in Section 5.1. Subsequently, in Section 5.2 we show by a bootstrap approach that the full problem can be approximated by (suitable corrections of) the homogeneous problem, where we separately consider three parts $I_1,I_2, I_3$ of the time interval *I*, each in a (sub)subsection.

A main challenge here is given by the fact that already in the homogeneous problem the evolution of the system is highly non-trivial, since the coefficients are time-dependent and potentially very large when considering frequencies $\eta$ which are very large (compared to $k^2$). We stress that this regime is essential, since any single echo chain stops after the last resonant time $\frac{\eta }{1}$. In particular, we stress that previous works [3, 5] included a **smallness constraint** of the form

5 $$\begin{aligned} \log (\eta ) \leqq C \end{aligned}$$

(see equation (10) in Section 3). Any size constraint on $\eta$, however, means that one can only deduce norm inflation up to *finite* time (not asymptotic behavior) and furthermore for finite frequencies all norms are equivalent. Therefore, under such a constraint it is *not* possible to distinguish between norm inflation with respect to different norms and there also is *no* blow-up. A key effort of this article thus lies in removing this constraint and studying the high frequency regime. We first show in Section 4 for a simplified model, that this is not only a technical challenge, but asymptotics indeed *qualitatively differ* in this regime and hence simpler methods of proof (such as in Section 3.1) necessarily fail.

We remark that Section 4 is not essential for the proof of our main result, but unlike the exact model considered in Section 5 allows us to construct explicit solutions (in terms hypergeometric functions and power laws) and hence to show in a more transparent way *why* at high frequencies we obtain a different exponent in the frequency dependence than might be expected from simpler heuristics. This model thus serves as an important preliminary step and heuristic, preceding the analysis of the full model (or the three mode model) in Section 5.2.

The remainder of our article is structured as follows:

- In Section 2 we show that for any $c \in {\mathbb {R}}$ the pair
  $$\begin{aligned} \omega _{\text {low}}(t,x,y)&=-1 + c \cos (x-ty), \\ v_{\text {low}}(t,x,y)&= (y,0) - \nabla ^{\perp } \frac{1}{1+t^2} c \cos (x-ty), \end{aligned}$$
  is a classical solution of the Euler equations (with infinite energy). While not stationary in Eulerian coordinates, we note that $\omega _{\text {low}}$ is stationary in coordinates $(x+ty,y)$. We call such a function a *traveling wave-like* solution. In particular, we observe that choosing *c* sufficiently small any small $H^{s}_{loc}$ (or even locally analytic) neighborhood of Couette flow contains traveling wave-like solutions. Our Theorem 1 then argues that the (simplified) linearization around such waves captures much more of the nonlinear dynamics than the linearization around Couette flow and in particular captures Gevrey norm inflation, “nonlinear” resonances (called fluid echoes) and blow-up. More precisely, our problem (4) then corresponds to a simplification of the linearized Euler equations around this state, where we drop a term involving $\frac{\sin (x)}{1+t^2}$, which we expect to be negligible. Section 2 serves to introduce this model, some changes of coordinates and to establish local and global well-posedness of the model (with highly sub-optimal exponential growth bounds).
- In Section 3 we introduce the echo chain mechanism for a model problem similar to the one considered in [3]. Furthermore, we show that under a smallness assumption (5) on $\eta$ and *c* similar to the one imposed in [3, 5], also the full model exhibits echo chains and norm inflation. We remark that this part of the article is self-contained and allows for a much shorter and simpler proof than the general case but crucially relies on the smallness assumption. Since this condition limits considerations to finite $\eta$ (where all norms are equivalent and norm inflation stops after a finite time), a key challenge and improvement of the this article is hence to remove this restriction in the following sections.
- In Section 4 we build an improved model which also takes into account the interaction of neighboring modes in the regime where $\eta$ is much larger than allowed by the smallness assumption. In particular, we show that the smallness restriction was not only a technical constraint, but that that the growth of solutions qualitatively differs in the large frequency regime and is connected to estimates on the 1D Schrödinger problem with scaling-critical potentials. The model character of the problem here allows for a more transparent discussion of the growth and decay properties of solutions on different time intervals and illustrates how their interaction leads to a modified dependence on the large frequency parameter.
- In Section 5 we then show that the full problem satisfies the same modified growth. In particular, we prove that for large frequencies $\eta$ with respect to *y* (compared to $c^{-1}$) the dependence on $\eta$ differs from the one in [3, 5] (it is given by a modified power law). Here Section 5.1 introduces the so-called *three mode model*, which is a restriction of the full model to the resonant mode and its neighbors at around the resonant time $t \approx \frac{\eta }{k}$. In Section 5.2 we then show that these three modes determine the growth of the full model during this time interval, which is the main part of our proof. In Section 5.3 we combine the growth on each time interval into a chain to build solutions which exhibit norm inflation. Furthermore, we construct solutions which are global in time and exhibit inviscid damping (that is convergence of the velocity) but whose vorticity asymptotically diverges.

We remark that in the high regularity, small $c,\eta$ regime the nonlinear problem and our model problem both asymptotically approach transport dynamics. It remains a challenging problem to determine how the full nonlinear Euler equations near Couette flow behave outside this regime and whether they also exhibit modified asymptotics similar to this linear model.

#### Notation

We use the following notational conventions:

- The spatial Fourier transform of a function $\omega (t, x, y) \in L^2 ({\mathbb {T}}\times {\mathbb {R}})$ is denoted by ${\tilde{\omega }}(t,k,\eta ) \in L^2({\mathbb {Z}}\times {\mathbb {R}})$.
- The Gevrey class ${\mathcal {G}}_{C,\frac{1}{s}}$ is defined in terms of the Fourier transform, that is $u \in {\mathcal {G}}_{C,\frac{1}{s}}$ if $\exp (C|\eta |^{s}){\tilde{u}} \in L^2$. Here, we omit the *k* dependence in the exponent since for the functions we consider only the region $|k|\leqq |\eta |$ is of interest.
- We use $a \lesssim b$ to denote that there exists an absolute constant $C>0$ such that $|a|\leqq C |b|$. In particular, we omit absolute value signs in our notation.
- In our calculations $c \in (0,0.2)$ and $\eta \in {\mathbb {R}}$ can be treated as arbitrary but fixed parameters. While the low frequency regime allows for rather simple arguments (see Section 3), in the high frequency regime where $|\eta |$ is much larger than $c^{-1}$ much finer control of cancellations is necessary.
- There we use $a \approx b$ to denote that there exists a constant $C_1>1$, such that $C_1^{-1} a\leqq b \leqq C_1 a$ such that $|C_{1}-1|\leqq 0.01$ for the regime of *c* and $\eta$ we are considering (with a possibly even smaller deviation for *c* smaller and/or $\eta$ larger). For example, in the regime of large $\eta$ it holds that $\eta ^{2-c}+\eta \approx \eta ^{2-c}$.

## Local Wellposedness and Asymptotic Stability

Our choice of the low-frequency vorticity $\omega _{\text {low}}(t,x,y)=-1+ c \cos (x+ty)$ (including Couette flow and Eulerian coordinates) is motivated by the Fourier coupling introduced by $\cos (x)$ and the fact that $\omega _{\text {low}}$ is a solution of the nonlinear Euler equations, which for small *c* can be considered as a very small analytic perturbation of Couette flow.

### Lemma 1

Let $c \in {\mathbb {R}}$ and define

$$\begin{aligned} \omega _{\text {low}}(t,x,y)&=-1 + c \cos (x-ty), \\ v_{\text {low}}(t,x,y)&= (y,0) - \nabla ^{\perp } \frac{1}{1+t^2} c \cos (x-ty). \end{aligned}$$

Then $(\omega _{\text {low}}, v_{\text {low}})$ is a classical solution of the Euler equations on ${\mathbb {T}}\times {\mathbb {R}}$ (with infinite energy) and $\omega (t,x+ty,y)$ does not depend on time.

In analogy to dispersive equations we call $\omega _{\text {low}}$ a *traveling wave*-like solution.

### Proof

In the study of stationary solutions to the Euler equations one observes that the equation

$$\begin{aligned} 0 = v \cdot \nabla \omega = \nabla ^{\perp } (-\varDelta )^{-1} \omega \cdot \nabla \omega \end{aligned}$$

implies that the gradients of the stream function $\phi =(-\varDelta )^{-1} \omega$ and of the vorticity are parallel. Therefore, they locally share level sets and locally one can express $\omega$ as a function of $\phi$. Conversely, if there exists a smooth function *f* such that $\omega =f(\phi )$, then by the chain rule $v\cdot \nabla \omega =0$ and one obtains a stationary solution (see for example [1]).

In the following we use the fact that $\cos (x-ty)$ is an eigenfunction of the the Laplacian and adapt this method to construct time-dependent solutions $\omega (t,x,y)=\omega (0, x-ty,y)$ which move with Couette flow. We observe that for every $t\in {\mathbb {R}}$ the stream function of the perturbation $\frac{1}{1+t^2} c \cos (x-ty)= (-\varDelta )^{-1} c \cos (x-ty)$ and the perturbation of the vorticity $c \cos (x-ty)$ have the same level sets (and are even colinear). Therefore the gradients are parallel and

$$\begin{aligned} \nabla ^{\perp } \frac{1}{1+t^2} c \cos (x-ty) \cdot \nabla \omega _{\text {low}} =0. \end{aligned}$$

Inserting this fact into the Euler equations it follows that

$$\begin{aligned} \partial _t\omega _{\text {low}} + v_{\text {low}} \cdot \nabla \omega _{\text {low}} = \partial _t\omega _{\text {low}} + y \partial _x \omega _{\text {low}} =0, \end{aligned}$$

which concludes the proof. $\quad \square$

In our equation (4) we now consider the linearization around $\omega _{\text {low}}$, where we omit the transport by $\frac{c\sin (x)}{1+t^2}\partial _y$ for simplicity. Since our equation (4) involves multiplication by a sine, it has an explicit characterization in Fourier variables:

$$\begin{aligned}&\partial _t{\tilde{\omega }}(t,k,\eta )+ \frac{c\eta }{(k-1)^2+(\eta -(k-1)t)^2} {\tilde{\omega }}(t,k-1,\eta )\\&\quad - \frac{c\eta }{(k+1)^2+(\eta -(k+1)t)^2} {\tilde{\omega }}(t,k+1,\eta )=0. \end{aligned}$$

In particular, we note that this equation decouples with respect to $\eta$ (while linearizations around shear flows instead decouple with respect to *k*). Hence, we may consider $\eta \in {\mathbb {R}}$ to be a given parameter and introduce a new time variable $\tau$:

6 $$\begin{aligned} t = \tau \eta . \end{aligned}$$

With respect to this variable (4) can be equivalently expressed as

7 $$\begin{aligned} \begin{aligned}&\partial _\tau \omega (\tau ,k,\eta ) + c \frac{1}{(k-1)^2} \frac{1}{\eta ^{-2} +(\frac{1}{k-1}-\tau )^2} \omega (\tau ,k-1,\eta ) \\&\quad - c \frac{1}{(k+1)^2} \frac{1}{\eta ^{-2} +(\frac{1}{k+1}-\tau )^2} \omega (\tau ,k+1,\eta )=0. \end{aligned} \end{aligned}$$

In particular, we note that in this formulation all *critical times* are given by $\frac{1}{j}$ for $j \in {\mathbb {N}}$ and thus independent of $\eta$. Furthermore, there are no critical times after time $\tau =1$, which implies that norm inflation or instability results are restricted to the evolution on small times and that the evolution is asymptotically stable after time $\tau =2$.

### Proposition 1

Let *X* be a weighted $L^2$ (or $l^2$) space on the Fourier side (e.g. a fractional Sobolev or Gevrey space) such that the weight $\rho (k,\eta )$ satisfies $\sup _{\eta } \frac{\rho (k\pm 1,\eta )}{\rho (k,\eta )}\leqq C_1 < \infty$. Then there exists a constant $C=C(cC_1)$ such that any solutions of (7) satisfy

$$\begin{aligned} \Vert \omega (\tau )\Vert _{X}\leqq C \Vert \omega (2)\Vert _{X} \end{aligned}$$

for all $\tau \geqq 2$. Furthermore, $\omega (\tau )$ strongly converges in *X* to a limit $\omega _{\infty } \in X$ as $t \rightarrow \infty$.

### Proof of Proposition 1

We may estimate the multipliers by

$$\begin{aligned}&\frac{c}{l^2} \frac{1}{\eta ^{-2} + (\frac{1}{l}-\tau )^2} \\&\quad \leqq c \frac{1}{0+(2-\frac{1}{l}+(\tau -2))^2} \\&\quad \leqq \frac{c}{(1+(\tau -2))^2}, \end{aligned}$$

irrespective of the size of $\eta$ or of $l \in {\mathbb {Z}}, l \ne 0$. We further remark that if $l \eta <0$ no restriction is necessary, since then there cannot be any cancellation. We may estimate

$$\begin{aligned} \partial _t\Vert \omega \Vert _{X}&\leqq \frac{c}{1+(\tau -2)^2}(\Vert \omega (k+1)\Vert _{X}+ \Vert \omega (k-1)\Vert _{X}) \\&\leqq \frac{2cC_1}{1+(\tau -2)^2} \Vert \omega \Vert _{X}. \end{aligned}$$

The result then immediately follows from integrating this inequality with

$$\begin{aligned} C:= \exp \left( 2cC_1 \Vert \frac{1}{1+(\tau -2)^2}\Vert _{L^1}\right) . \end{aligned}$$

$$\square $$

We remark that the admissible class of spaces *X* includes fractional Sobolev spaces $H^s$ and Gevrey spaces ${\mathcal {G}}_{C,\frac{1}{s}}$, but not the homogeneous fractional Sobolev spaces due to the quotient $\frac{|\eta \pm 1|^s}{|\eta |^s}$ degenerating for $\eta \downarrow 0$.

In order to analyze possible norm inflation, in the following we hence focus on characterizing the evolution on the time interval [0, 2]. Here, as a first easy, non-optimal estimate, we may roughly control the multiplier by

$$\begin{aligned} \sup \limits _{l \ne 0} \frac{|c\eta ^2|}{l^2}= c\eta ^2, \end{aligned}$$

and thus obtain

### Theorem 2

Let *X* be as in Proposition 1. Then if the initial data is localized on the mode $\eta$, it holds that

$$\begin{aligned} \Vert \omega (\tau )\Vert _{X} \leqq \exp (2C_1 c\eta ^{2} \min (2,\tau )) \Vert \omega _0\Vert _{X}, \end{aligned}$$

for all $\tau \geqq 0$. Furthermore, $\omega (\tau )$ strongly converges in *X* as $\tau \rightarrow \infty$.

### Proof of Theorem 2

By Proposition 1 it suffices to establish a bound for $\tau \leqq 2$. Here, we estimate the Fourier multiplier in (7) by $c \eta ^2$ and thus obtain that, for $\omega _0$ localized at frequency $\eta$, it holds that

$$\begin{aligned} \Vert \partial _\tau \omega \Vert _{X} \leqq&c \eta ^2 (\Vert S_{+1} \omega \Vert _{X}+ \Vert S_{-1} \omega \Vert _{X}) \\ \leqq&2 C_1 c \eta ^2 \Vert \omega \Vert _{X}, \end{aligned}$$

where $S_{\pm 1}$ denotes a shift in the *k* dependence. The result then follows by Gronwall’s lemma. $\quad \square$

We stress that these estimates are far from optimal. However, they allow us to control the evolution until a small positive time $\tau _0>0$, after which a more detailed study of resonance chains establishes sharper bounds.

Indeed, in Section 5, we show that this linearized model attains the same Gevrey 2 class norm inflation results as the nonlinear problem around Couette flow considered in [3, 5]. Furthermore, the solutions exist globally in time and exhibit modified scattering and damping.

In order to introduce ideas, in the following Section 3 we consider the setting where *c* is very small compared to $\eta ^{-1}$. This setting allows for highly simplified proofs and serves to introduce the resonance mechanism in a clear way. In Section 4 we then introduce an improved “toy model”, which includes further cancellation mechanisms compared to the one studied in [3] and shows what modifications to asymptotic convergence should be expected for large $\eta$ (compared to $c^{-1}$). The results of Section 5 then show that the full linear problem exhibits the same kind of growth as this model and we further discuss implications for scattering and damping.

## Echoes, Paths and Norm Inflation

Theorem 2 of the preceding section proves wellposedness of the evolution equation for frequency localized initial data, but provides a very rough upper bound on the possible growth. In this section we study the evolution of equation (7)

8 $$\begin{aligned} \begin{aligned}&\partial _\tau \omega (\tau ,k,\eta ) + c \frac{1}{(k-1)^2} \frac{1}{\eta ^{-2} +(\tau - \frac{1}{k-1})^2} \omega (\tau ,k-1,\eta ) \\&\quad - c \frac{1}{(k+1)^2} \frac{1}{\eta ^{-2} +(\tau -\frac{1}{k+1})^2} \omega (\tau ,k+1,\eta )=0. \end{aligned} \end{aligned}$$

for $0\leqq \tau \leqq 2$ in more detail in order to obtain a finer description of the associated norm-inflation mechanism. More precisely, in this section we consider the setting where $\eta$ satisfies a smallness assumption (10) as in [3, 5], which allows for a short and simple proof of norm inflation. We remark that this section is self-contained and that the low frequency results of this section are not required for our main results. It is instead intended to provide a simple introduction to the main growth mechanism and to motivate why removing the smallness assumption is a highly non-trivial, challenging problem.

In order to develop a heuristic for the growth mechanism, formally consider the equation with $\omega (\tau , k-1,\eta )$, $\omega (\tau , k+1,\eta )$ frozen in time. Then integrating the equation we obtain that, for two times $\tau _0<\tau _1$, it holds that

$$\begin{aligned}&\omega (\tau _1,k,\eta )-\omega (\tau _0,k,\eta ) \\&\quad \approx \omega (\tau _0, k-1,\eta ) \int _{\tau _0}^{\tau _1} c \frac{1}{(k-1)^2} \frac{1}{\eta ^{-2} +(\tau - \frac{1}{k-1})^2} \, \mathrm{d}\tau \\&\qquad - \omega (\tau _0, k+1,\eta ) \int _{\tau _0}^{\tau _1} c \frac{1}{(k+1)^2} \frac{1}{\eta ^{-2} +(\tau - \frac{1}{k+1})^2} \, \mathrm{d}\tau . \end{aligned}$$

In reference to fixed point iterations, we also call this heuristic the first Duhamel iteration (which inserts the initial datum into the fixed point map. See also Section 3 for higher iterations.). If $\tau _0$ and $\tau _1$ are suitably chosen the growth factor can thus be expected to be comparable to

$$\begin{aligned} \frac{1}{(k-1)^2} \int _{-\infty }^\infty \frac{c}{\eta ^{-2}+(\tau -\frac{1}{k-1})^2} \, \mathrm{d}\tau = c\pi \eta \frac{1}{(k-1)^2} = c \pi \frac{\eta }{(k-1)^2}. \end{aligned}$$

Similarly to the nonlinear equations studied in [3, 5], the main norm inflation mechanism of (7) is then for mode $(k,\eta )$ to induce growth of the mode $(k-1,\eta )$ at time $\tau \approx \frac{1}{k}$, which in turn induces growth of the mode $(k-2,\eta )$ at time $\tau \approx \frac{1}{k-1}$. Iterating this heuristic along a chain until time $\tau \approx 1$, this suggests a total growth factor of

9 $$\begin{aligned} \frac{\eta }{k^2} \frac{\eta }{(k-1)^2} \cdots \frac{\eta }{1^2}= \frac{\eta ^{k}}{(k!)^2}. \end{aligned}$$

This factor achieves its maximum with respect to *k* for $k \approx \sqrt{\eta }$, which yields $\exp (C \sqrt{\eta })$ and thus a norm inflation result consistent with Gevrey 2 regularity. While single echoes or small numbers of echoes are well-studied in the physical and numerical literature [11, 13] we stress here that long chains of echoes correspond to Gevrey regularity. Indeed, one of the main challenges in the following will be to construct infinitely many chains of arbitrarily long lengths in such a way that the evolution can be controlled for all times.

Compared to the nonlinear results of [5], we highlight the following differences of the present setting:

- Due to the difficulties involved in controlling a nonlinear evolution around growing perturbations, [5] establishes such a growth chain until time $t \approx \frac{1}{2k}\approx \frac{1}{2\sqrt{\eta }}\ll 1$. Our results established for the linear equation (7) instead capture a full echo chain as well as the subsequent asymptotic stability as $\tau \rightarrow \infty$.
- The more explicit structure of the associated Duhamel iteration scheme allows us to establish more precise upper and lower bounds and in particular quantify the dependence on the parameter *c*, i.e. the size of the low-frequency part. Furthermore, as we discuss in Section 4, the evolution for $\eta$ much larger than $k^2$ introduces additional logarithmic corrections, which we show in Section 5 to result in a correction to the asymptotic evolution. In particular, prior works included a constraint of the form
  $$\begin{aligned} c\log (1+|\eta |)\lesssim 1, \end{aligned}$$
  where a change in exponent by *c* is then comparable to multiplication by a constant.
- Identifying the sharp growth behavior and removing this constraint allows us to construct families of initial data, for which the velocity field converges while the vorticity diverges (see Section 5)! We view this as an indication that nonlinear inviscid damping, understood as asymptotic convergence of the velocity field, might still hold in lower regularity despite norm inflation results for the vorticity.

As a first step, in the following theorems we improve the bounds by $\exp (C \eta ^2)$ to Gevrey 2-type estimates of the above form. Similarly to [2, 5] in this section we impose a logarithmic smallness condition

10 $$\begin{aligned} c \leqq C \ln (|\eta |)^{-1}, \end{aligned}$$

which greatly simplifies this proof. In particular, it allows us to clearly demonstrate the growth mechanism, obtain explicit (approximate) solutions and point out where these methods of proof would fail for high frequencies.

We stress that for any fixed *c* this smallness condition prevents us from considering (sequences of) arbitrarily large $\eta$, which is necessary to establish instability and modified scattering results. A key effort of this article in Sections 4 and 5.2 is to remove this constraint. Here, it turns out that large frequencies not only pose technical challenges, but that for larger $\eta$ the asymptotics indeed differ. In Section 5.3 we show that taking into account these modified asymptotics our linear system exhibits norm inflation and echo chains for all $\eta$, as well as modified scattering.

### Theorem 3

(Resonance chain) Let $\eta >1$, $l \in {\mathbb {N}}$ with $\frac{\eta }{l^2}\geqq 1$ and let $0<c\leqq \min ((\ln (1+\eta ^2))^{-1}, \frac{1}{100})$. Let further $\tau _0=\frac{1}{2}(\frac{1}{l+1}+\frac{1}{l})$ and $\tau _1=1.5$. Then the solution $\omega (\tau )$ of (7) with

$$\begin{aligned} \omega (\tau _0,k)=\delta _{k,l} \end{aligned}$$

satisfies

$$\begin{aligned} \omega (\tau _1,k)&\geqq c^{l} \left( 1-\frac{c}{1-c}\right) ^l \frac{\eta ^{l}}{(l!)^2} \text { if } k \in \{1,3\}, \\ \omega (\tau _1,k)&\leqq c^{l} \left( 1+\frac{c}{1-c}\right) ^l \frac{\eta ^{l}}{(l!)^2} \text { if } k \in \{1,3\}, \\ |\omega (\tau _1,k)|&\leqq c^{|k-l|} c^{l} \left( 1+\frac{c}{1-c}\right) ^l \frac{\eta ^{l}}{(l!)^2} \text { if }k \not \in \{1,3\} . \end{aligned}$$

Furthermore, there exists $\omega _{\infty }(k)$ such that $\omega (t,k)\rightarrow \omega _{\infty }(k)$ as $\tau \rightarrow \infty$ and $\Vert \omega _{\infty }\Vert _{X}\leqq C_X \Vert \omega (\tau _1)\Vert _{X}$. In particular, the associated velocity field converges as $\tau \rightarrow \infty$.

We remark that such a result also yields bounds for frequency-localized initial data globally in time by using Theorem 2 to control the evolution until time $\tau _0$. In order to prove Theorem 3 we use precise description of the evolution operator around single resonances $\tau \approx \frac{1}{k}$ for $k=l,l-1, \ldots , 1$, which we then combine to establish the over all growth.

### Single Resonance Estimates

We note that in equation (7) modes only directly interact with their nearest neighbors. Thus, if we prescribe that $\omega (\tau _0)=e^{ik_0 x +i \eta y}$, the value of ${\tilde{\omega }}(\tau _1,k,\eta )$ involves sequences $\gamma =(k_0,k_0-1,\ldots , k)$, where a mode $\gamma _i$ influences a mode $\gamma _{i+1}$ by nearest neighbor interaction. Indeed, any finite Duhamel iteration can be associated with a sum over all such sequences with an upper bound on their length and integrals of the form

11 $$\begin{aligned} \iint _{\tau _0 \leqq s_1 \leqq s_2 \leqq \cdots \leqq \tau _1} \prod _{i=1}^{|\gamma |}\frac{1}{\gamma _i^2}\frac{c}{\eta ^{-2} +(s_i-\frac{1}{\gamma _i})^2}\, \mathrm{d}s_{i}. \end{aligned}$$

In order to simplify discussion, we introduce some notation.

#### Definition 1

Let $k,k_0 \in {\mathbb {Z}}$ with $\text {sgn}(k)=\text {sgn}(k_0)$. Then a *path* $\gamma$ from $k_0$ to *k* is a finite sequence $\gamma =(\gamma _i)_{i=1}^n$ with $\gamma _1=k_0, \gamma _n=k$ and $|\gamma _{i+1}-\gamma _i|=1$. We call the integral (11) the *integral* associated to $\gamma$, $I[\gamma ]$ and call $n-1=:|\gamma |$ the *length* of $\gamma$.

We remark that equation (7) introduces a post-multiplication by $\sin (x)$ and a single Duhamel iteration hence results in paths $(k_0,k_0+1)$ and $(k_0,k_0-1)$, which we thus denote as paths of length 1.

Given a time interval $\frac{1}{l+1}< \tau _0< \frac{1}{l}< \tau _1 < \frac{1}{l-1}$, we observe that integrals tend to be small unless $\gamma _i=l$ for some *i*. Indeed, if $\gamma _i \ne l$, then $|\frac{1}{\gamma _i}-\frac{1}{l}| \geqq \frac{c}{l^2}$ and we may estimate each integral with respect to $s_i$ by

12 $$\begin{aligned} \begin{aligned}&\frac{1}{l^2} \int _{|\tau -\frac{1}{l}|\geqq \frac{1}{2l(l-1)}} \frac{c}{\eta ^{-2}+(\tau -\frac{1}{l})^2} \, \mathrm{d}\tau \\&\quad \leqq \frac{1}{l^2} \int _{s\geqq \frac{1}{2l(l-1)}} \frac{c}{\tau ^2} \, \mathrm{d}\tau \leqq 4 c, \end{aligned} \end{aligned}$$

uniformly in $\eta$ and in *l*.

#### Definition 2

Let $l \in {\mathbb {N}}$ and define $T_0:=\frac{1}{2}(\frac{1}{l+1}+\frac{1}{l})< \frac{1}{l}<\frac{1}{2}(\frac{1}{l-1}+\frac{1}{l})=:T_1$. Let further $k, k_0 \in {\mathbb {N}}$ and let $\gamma$ be a path from $k_0$ to *k*. We call $\gamma _i$ *resonant* if $\gamma _i=l$ and *non-resonant* else. A path $\gamma$ is called *non-resonant* if $\gamma _i$ is non-resonant for all *i*.

#### Proposition 2

(Single resonance bound) Let $l \in {\mathbb {N}}$, $\eta >0$ and let

$$\begin{aligned} \tau _0:=\frac{1}{2}\left( \frac{1}{l+1}+\frac{1}{l}\right)<\frac{1}{l}< \frac{1}{2}\left( \frac{1}{l}+\frac{1}{l-1}\right) =: \tau _1, \end{aligned}$$

and suppose that the constant $c>0$ is sufficiently small that $2c \leqq \ln (1+ \eta )^{-1}$.

1. Let $k_0 \in {\mathbb {N}}, k_0\ne l$ and let $\omega (\tau , \cdot )$ be the solution of (7) with
  $$\begin{aligned} \omega (\tau _0,k)= \delta _{k, k_0}. \end{aligned}$$
  Then it holds that
  $$\begin{aligned} |\omega (\tau _1,k)- \omega (\tau _0,k)| \leqq C (c^{|k-l|+|k_0-l|} \frac{\eta }{l^2} + c^{|k-k_0|}) \end{aligned}$$
  for all $k \in {\mathbb {N}}$. Here, with slight abuse of notation $|k-l|=\max (|k-l|,2)$ denotes the length of the shortest path connecting *k* and *l*. This agrees with the absolute value if $k\ne l$, but is 2 if $l=k$, since the shortest path $(k,k\pm 1, k)$ has length 2.
2. Let $\omega (\tau ,\cdot )$ be the solution of (7) with
  $$\begin{aligned} \omega (\tau _0,k)= \delta _{k,l}. \end{aligned}$$
  Then it holds that
  $$\begin{aligned} \omega (\tau _1,l\pm 1)&\approx \frac{\eta }{l^2}, \\ |\omega (\tau _1,l)-1|&\leqq C c^2 \frac{\eta }{l^2}, \\ |\omega (\tau _1,k)|&\leqq C (c^{|k-l|}\left( 1+\frac{\eta }{l^2})\right) \text { else}. \end{aligned}$$

#### Proof of Proposition 2

We argue by a Duhamel-iteration approach and summing over all paths starting in $k_0$ and ending in *k*. Here, we first note that if a path is non-resonant, we may estimate its contribution by $c^{|\gamma |}$. If we then estimate the number of such paths from above by $2^{|\gamma |}$, the contribution by all non-resonant paths is bounded by

$$\begin{aligned} \sum _{|\gamma |\geqq \text {dist}} (2c)^{|\gamma |} = \frac{1}{1-2c} (2c)^{\text {dist}}, \end{aligned}$$

where dist is the length of the shortest (non-resonant) path connecting *k* and $k_0$. Our main challenge in the following is thus going to be to control resonances and in particular the interaction between multiple resonances (that is, $\gamma _i=l$ for several indices *i*).

*Ad 1* Let $k_0 \ne l$ be given and let $k \in {\mathbb {N}}$. Let further $\gamma$ be a path starting in $k_0$ and ending in *k* and denote $j=|\gamma |$. We have already discussed the case of all non-resonant paths above, so suppose that $\gamma _i=l$ for some *i* and let $(i_\kappa )_{\kappa =1}^n$ denote all such indices. We note that we first have to reach *l* starting from $k_0$ and thus $i_1\geqq |k_0-l|$. Similarly it follows that $|i_n-j|\leqq |k-l|$. Furthermore, $i_{\kappa }\leqq i_{\kappa +1}-2$, since consecutive entries in a path are distinct.

In order to bound $I[\gamma ]$, first consider two subsequent resonances indices $i_{\kappa }$ and $i_{\kappa +1}$. Keeping these indices fixed, we first integrate over the intermediate indices $s^1, \ldots s^{l}$ and consider

$$\begin{aligned} \int _{s_{i_\kappa }\leqq s^1\leqq \cdots \leqq s^{l}\leqq s_{i_{\kappa +1}}} \prod _{j=1}^{l}\frac{1}{\gamma _j^2} \frac{c}{\eta ^{-2}+\left( s^j-\frac{1}{\gamma _j}\right) ^2} \,\mathrm{d}s_j \leqq (Cc)^l |s_{i_{\kappa +1}}-s_{i_\kappa }|, \end{aligned}$$

where we used that by assumption all these $\gamma _{j}\ne l$ are non-resonant and can be bounded by powers of *c* and bounded one of the integrals by $c (s_{i_{\kappa +1}}-s_{i_\kappa })$.

Repeating this argument for all $\kappa$, we only need to consider the integrals with respect to the resonances:

$$\begin{aligned} (cC)^{j} \iint _{T_0\leqq s_{i_1}\leqq s_{i_2}\leqq \cdots \leqq s_{i_n}\leqq T_1} \frac{1}{\eta ^{-2}+\left( s_{i_n}-\frac{1}{l}\right) ^2} \prod _{\kappa =1}^{n-1}\frac{s_{i_{\kappa +1}}-s_{i_\kappa }}{\eta ^{-2} +\left( s_{i_\kappa }-\frac{1}{l}\right) ^2}. \end{aligned}$$

Rescaling and shifting all $s_{\cdot }$ as $\eta ^{-1}(s-\frac{1}{l})$, we obtain a factor $\eta ^{-j}$ from the Jacobian, a factor $\eta ^{-j+1}$ from the linear factors, and a factor $\eta ^{2j}$ from the denominators, and thus, in total:

$$\begin{aligned} (cC)^{j} \eta \iint _{\eta \left( T_0-\frac{1}{l}\right) \leqq s_{i_1}\leqq s_{i_2}\leqq \cdots \leqq s_{i_n}\leqq \eta \left( T_1-\frac{1}{l}\right) } \frac{1}{1+s_{i_n}^2} \prod _{\kappa =1}^{n-1}\frac{s_{i_{\kappa +1}}-s_{i_\kappa }}{1+s_{i_\kappa }^2}. \end{aligned}$$

It remains to estimate the integral over this now $\eta$ dependent region.

Expanding the product in the numerator and looking at each summand separately we need to consider monomials in the numerator. If a factor $s_{i_\kappa }$ does not appear in the numerator we simply bound by

$$\begin{aligned} \int _{{\mathbb {R}}} \frac{1}{1+s_{i_\kappa }^2}=\pi . \end{aligned}$$

If it appears once, we may compute

$$\begin{aligned} \int _{s} \frac{|s|}{1+s^2}=\frac{1}{2}\ln \left( 1+s^2\right) +c. \end{aligned}$$

If it appears twice, we bound

$$\begin{aligned} \int _{s_{i_{\kappa -1}}\leqq s_{i_\kappa }\leqq s_{i_{\kappa +1}}} \frac{s_{i_{\kappa }}^2}{1+s_{i_\kappa }^2} \leqq s_{i_{\kappa +1}-s_{i_{\kappa -1}}}, \end{aligned}$$

but in this way $i_{\kappa }$ does not appear in the integral anymore and we can repeat the above argument with this resonance removed. Hence, we only need to consider monomials where each $s_{i_\kappa }$ appears either to power 1 or does not appear:

$$\begin{aligned} \iint _{\eta \left( T_0-\frac{1}{l}\right) \leqq s_{i_1}\leqq s_{i_2}\leqq \cdots \leqq s_{i_n}\leqq \eta \left( T_1-\frac{1}{l}\right) } \prod _{i_{\kappa } \text { appears}} \frac{|s_{i_{\kappa }}|}{1+s_{i_\kappa }^2} \prod _{i_{\kappa } \text { does not}} \frac{1}{1+s_{i_\kappa }^2}. \end{aligned}$$

As discussed above the integrals over the “does not” case can be bounded by $\pi ^{j_2}$, where $j_2=j-j_1$ denotes the number of such cases. The integral over the first product is bounded by a power of a logarithm:

13 $$\begin{aligned}&\frac{1}{j_1!} \left( \frac{1}{2}\ln \left( 1+\left( \eta \left( T_1-\frac{1}{l}\right) \right) ^2\right) +\frac{1}{2}\ln \left( 1+\left( \eta \left( T_0-\frac{1}{l}\right) \right) ^2\right) \right) ^{j_1} \nonumber \\&\approx \frac{1}{j_1!} \left( C\ln \left( 1+\frac{\eta }{l^2}\right) \right) ^{j_1}. \end{aligned}$$

Recalling the additional prefactor $c^{|\gamma |}$, summing over all such $j_1$ then leads to a bound of this contribution by

$$\begin{aligned} \left( 1+\frac{\eta }{l^2}\right) ^{Cc} = \exp \left( Cc\ln \left( 1+\frac{\eta }{l^2}\right) \right) \leqq \exp ({\tilde{C}}), \end{aligned}$$

where we used the logarithmic smallness assumption

$$\begin{aligned} c\ln \left( 1+\frac{\eta }{l^2}\right) \ll 1. \end{aligned}$$

Hence, this exponential can be treated as a perturbation provided *c* and thus ${\tilde{C}}$ is sufficiently small.

*Ad 2* We proceed similarly as in case 1, but note that

$$\begin{aligned} \int _{\tau _0}^{\tau _1} \frac{1}{l^2}\frac{c}{\eta ^{-2}+\left( s-\frac{1}{l}\right) ^2} \approx c \frac{\eta }{l^2}. \end{aligned}$$

All longer paths starting in *l* and ending in $l- 1$ have length at least 3 and hence the sum over all these paths can again be controlled by

$$\begin{aligned} \frac{c^2}{1-c} \frac{c \eta }{l^2}. \end{aligned}$$

We hence obtain the claimed comparability with factors $1\pm \frac{c^2}{1-c}$. For $k=l$, we note that the shortest non-trivial path has length 2 and for $k\not \in {l-1,l,l+1}$ we argue as before except that all paths start in $\gamma _1=l$. $\quad \square$

We remark that by using the linearity of the problem this further yields a convolution-type bound for $\omega (\tau _0)$ not being localized on a single mode. In the following we then combine these mode-wise upper and lower bounds as well as the local wellposedness of Theorem 2 to construct an explicit example of a function that exhibits the Gevrey norm inflation up to time $\tau =1$ and afterwards is asymptotically stable and hence also exhibits inviscid damping. In Section 5 we show with considerable technical effort that a similar but distinct result also hold if $\eta$ does not satisfy a logarithmic bound.

### Proof of Theorems 3

Using the single-resonance results of Proposition 2 we are now ready to prove Theorem 3.

We proceed in multiple steps:

1. We use local well-posedness to control the evolution from time $\tau =0$ up to a small positive time $\tau ^*=\tau ^*(c,\eta )\ll 1$.
2. We next choose $l \in {\mathbb {N}}$ such that $\frac{1}{l}>\tau ^*$ and *l* is maximal with this property and prescribe $\omega (\tau )$ at the time $\tau _0=\frac{1}{2}(\frac{1}{l+1}+\frac{1}{l})<\frac{1}{l}$ to be localized at frequencies $(l,\eta )$. Proposition 2 then allows us to control the evolution up to time $\tau _1=\frac{1}{2}(\frac{1}{l}+\frac{1}{l-1})>\frac{1}{l}$, where we in particular obtain upper and lower bounds on the modes $l\pm 1$ and upper bounds on all other modes.
3. We then iterate this control another $l-1$ times and establish upper bounds on all modes and lower bounds along our chain of resonances $(l,l-1,l-2,\ldots , 1)$ until after time $\tau =1$.
4. By our construction of the coordinate $\tau$, after this time no resonances appear anymore and we may use Proposition 1 to control the long-time asymptotic behavior.

#### Proof of Theorem 3

We note that it suffices to establish upper and lower bounds on $\omega$ until time $\tau =1.5$, since after that time asymptotic stability and convergence of the velocity field follow by Proposition 1.

Let thus $l \in {\mathbb {N}}$ be given, to be fixed later. By Theorem 2 we may find initial data such that at time $\tau _l:=\frac{1}{2}(\frac{1}{l-1}+\frac{1}{l})$ it holds that

14 $$\begin{aligned} |\omega (\tau _l,l)| \geqq 0.5 \max |\omega (\tau _l)|=: 0.5 \theta . \end{aligned}$$

We then apply Proposition 2 using the linearity and a triangle inequality to obtain a convolution bound. That is, for $k \not \in \{l-1,l,l+1\}$, we obtain that

$$\begin{aligned} |\omega (\tau _{l+1},k)-\omega (\tau _{l},k)|&\leqq C \left( c^{|k-l|}\left( 1+\frac{\eta }{l^2}\right) \right) |{\tilde{\omega }}(\tau _l,l)|\\&\quad +\sum _{k_0\ne l} |{\tilde{\omega }}(\tau _l,k_0)| C \left( c^{|k-l|+|k_0-l|} \frac{\eta }{l^2} + c^{|k-k_0|}\right) \\&\leqq \theta C \left( c^{|k-l|}\left( 1+\frac{\eta }{l^2}\right) \right) + \theta C c^{|k-l|}\frac{1}{1-c}\frac{\eta }{l^2} +\frac{\theta }{1-c}. \end{aligned}$$

If $k=l$, the first term is replaced by

$$\begin{aligned} Cc^2 \frac{\eta }{l^2}. \end{aligned}$$

Finally, if $k\in \{l-1,l+1\}$, we obtain that

$$\begin{aligned} |\omega (\tau _{l+1},k)\mp C_{\pm } c\frac{\eta }{l^2} \omega (\tau _l,l) - \omega (\tau _{l},k)| \leqq \theta C c^{|k-l|}\frac{1}{1-c}\frac{\eta }{l^2} +\frac{\theta }{1-c}, \end{aligned}$$

where $C_{\pm 1}\approx 1$. Recalling that $\omega (\tau _l,l)$ achieves $\theta$ within a factor 2 and using that $c\frac{\eta }{l^2}\gg 1$, if follows that

$$\begin{aligned} \pm c\frac{\eta }{l^2} \omega (\tau _l,l) \approx \omega (\tau _{l+1},l \pm 1) \geqq 0.5 \max |\omega (\tau _{l+1})|. \end{aligned}$$

also achieves the new maximum within a factor 2 and is larger than the previous maximum by a factor $c\frac{\eta }{l^2}$.

We may thus repeat our application of Proposition 2 iteratively until for $k=1$, we obtain that

$$\begin{aligned} \omega (\tau _1,1)\approx \frac{c^{l}\eta ^{l}}{(l!)^2} \omega (\tau _l,l) \end{aligned}$$

achieves the full growth along a chain and again is comparable to the supremum at that time. $\quad \square$

While this result is quite useful and shows the growth mechanism, the logarithmic bound

$$\begin{aligned} c \ln \left( 1+\frac{\eta }{l^2}\right) \ll 1, \end{aligned}$$

prevents us from considering $\eta$ arbitrarily large. In particular, reverting the change of variables $t \mapsto \tau$, this means that after a final time $t=\max \eta$ there are no more resonances and the evolution is asymptotically stable. Furthermore, since we can only consider a finite set in $\eta$ all norms are equivalent and thus the associated growth while suggestive of a Gevrey regularity class is consistent with any norm.

We remark that also [3, 5] contain a similar constraint. While one might at first hope that this is a purely technical constraint, in our proof we saw various logarithmic terms appearing, where in particular the contribution by the path $\gamma =(l,l-1,l,l-1)$ is also bounded below. It is hence a large, non-negligible correction, which can not be treated perturbatively. Thus, our main goal in the following is to understand how the dynamics change and to remove this restriction. To this end, in Section 4 we first introduce an improved model problem and new methods of proof, which allow us to consider $\eta$ arbitrarily large. Then in Section 5 we show that this behavior also holds in the full problem.

## An Improved Model Problem

In order to better study the effect of resonances and, in particular, the behavior for large $\eta$ and/or large *c*, in the following we consider an abridged model which considers the evolution for $\frac{1}{l+1}< \tau < \frac{1}{l-1}$ and only considers the resonant mode *l* and its neighbor $l-1$:

$$\begin{aligned}&\partial _{\tau } \omega (\tau ,l-1)- \frac{c}{\eta ^{-2}+(\tau -\frac{1}{l})^2} \frac{1}{l^2} \omega (\tau ,l)=0, \\&\partial _{\tau } \omega (\tau ,l)+ \frac{c}{\eta ^{-2}+(\tau -\frac{1}{l-1})^2} \frac{1}{(l-1)^2} \omega (\tau ,l-1)=0. \end{aligned}$$

As remarked in Section 1.1, this section is not strictly necessary for the proof of our main result in Section 5. However, its model structure allows us to explain why the behavior at large frequencies necessarily strongly differs and how growth and decay on several time intervals connect to each other to yield a modified power law growth. Indeed in Section 5 we show that also the full model behaves similar to this two-mode model (or more accurately like the three-mode model involving the modes $l+1,l,l-1$), where our strategy of proof closely mirrors the one of Proposition 3 of this section (with each step of the proof now corresponding to a subsection). This section hence serves as an important preliminary step, highlighting the main challenges and illustrating the effects of large frequencies violating the smallness constraint.

The present model is very similar to the one considered in [3], except that our model does not estimate the coefficients by absolute values but rather keeps the signs, which corresponds to exploiting the real-valuedness of $\sin (x)$ (and hence anti-symmetry of the imaginary parts of the Fourier coefficients). We further remark that a similar model for a single echo has been studied in [11] both analytically and numerically, but not for chains and only in the small parameter regime.

As we will see in the following this yields important cancellation properties and further exposes a connection of this problem to the 1D Schrödinger problem with scaling critical potential.

As a simplification of calculations, we use that $\frac{1}{l-1}$ is non-resonant and approximate $\tau - \frac{1}{l-l}\approx \frac{1}{l^2}$ and thus consider the following *two-mode system*:

15 $$\begin{aligned} \begin{aligned}&\partial _\tau u - \frac{c}{\eta ^{-2}+\tau ^2} \frac{1}{l^2} v(\tau ) =0, \\&\partial _\tau v + \frac{c}{\eta ^{-2}+l^{-4}} \frac{1}{l^2} u(\tau ) =0. \end{aligned} \end{aligned}$$

Here we also shifted the time to $\tau \in [-l^{-2}, l^{-2}]$ for notational convenience.

In order to introduce ideas, we first consider the case where $\eta$ is not larger than $l^2$. There, we approximate $\eta ^{-2}+\tau ^2 \approx \eta ^{-2}+l^{-4}\approx \eta ^{-2}$ and obtain the following lemma:

### Lemma 2

(Small $\eta$ case) Let $\eta >0, l \in {\mathbb {Z}}$ and $c \in {\mathbb {R}}$ be given and consider the following approximation to system (15):

16 $$\begin{aligned} \begin{aligned} \partial _{\tau } \begin{pmatrix} u \\ v \end{pmatrix} + \begin{pmatrix} 0 &{}\quad -c\frac{\eta ^2}{l^2} \\ c \frac{\eta ^2}{l^2} &{}\quad 0 \end{pmatrix} \begin{pmatrix} u \\ v \end{pmatrix} =0, \end{aligned} \end{aligned}$$

for $\tau \in (-l^{-2}, l^{-2})$. Then, the unique solution is given by

17 $$\begin{aligned} \begin{pmatrix} u(\tau ) \\ v(\tau ) \end{pmatrix} = \begin{pmatrix} \cos \left( c \frac{\eta }{l^2} \tau \right) &{}\quad \sin \left( c \frac{\eta }{l^2} \tau \right) \\ -\sin \left( c \frac{\eta }{l^2} \tau \right) &{}\quad \cos \left( c \frac{\eta }{l^2} \tau \right) \\ \end{pmatrix} \begin{pmatrix} u(0) \\ v(0) \end{pmatrix}. \end{aligned}$$

In particular, it follows that $|u(\tau )|^2+|v(\tau )|^2$ is a conserved quantity.

### Proof

While the solution follows immediately by computation of the matrix exponential, for later reference we note an alternative proof arguing at the level of second derivatives. Formally differentiating the equation for *u* by $\tau$ and using the second equation, we obtain

$$\begin{aligned} \partial _\tau ^2 u + c^2\frac{\eta ^{4}}{l^4} u =0, \end{aligned}$$

which has a general solution

$$\begin{aligned} \alpha \cos \left( c \frac{\eta }{l^2} \tau \right) + \beta \sin \left( c \frac{\eta }{l^2} \tau \right) \end{aligned}$$

for constants $\alpha , \beta \in {\mathbb {R}}$. The first-order equation

$$\begin{aligned} \partial _\tau u - c \frac{\eta }{l^2} v =0 \end{aligned}$$

then further determines $\partial _\tau u$ and allows us to determine $\alpha$ and $\beta$ in terms of *u*(0), *v*(0). $\quad \square$

Having discussed the case of small $\eta$, in the following we are interested in the setting where $\eta \geqq l^2$ is potentially very large. Here, we may again consider decoupled second order equations instead of a system of first order equations:

18 $$\begin{aligned} \begin{aligned}&\partial _{\tau }^2 u + \frac{c^2}{\eta ^{-2}+ \tau ^2} u =0, \\&\partial _\tau (\eta ^{-2}+\tau ^2)\partial _\tau v + c^2 v =0. \end{aligned} \end{aligned}$$

We note that the equation for *u* is given by a stationary Schrödinger equation with potential $\frac{c^2}{\eta ^{-2}+ \tau ^2}$, which is a mollified version of the scaling critical potential $\frac{c^2}{\tau ^2}$.

The equation satisfied by *v* instead is a degenerate elliptic problem. However, we note that (15) allows us to determine *v* in terms of $\partial _\tau u$. Hence, in the following it suffices to study the evolution of *u*.

In Section 3 we showed that a Duhamel iteration converges, with the dominant terms being given by the nearest neighbor paths $(l,l-1,l,l-1,l, \ldots )$ of this section, but had to require that *c* is sufficiently small to control logarithmic corrections. The aim in what follows is to study the system (15) and show that for large $\eta$ better estimates hold and that only an absolute bound on *c* is required.

We recall that (18) is posed on the interval $(-l^{-2}, l^{-2})$. We may rescale our time variable as $\tau =l^{-2} t$, so that $t \in (-1,1)$ and obtain

19 $$\begin{aligned} \partial _{t}^2 u + \frac{c^2}{\eta ^{-2}l^4+t^2} u =0. \end{aligned}$$

We stress that this equation depends only on *c* and $\frac{\eta }{l^2}$, but not on $\eta$ and *l* separately. For simplicity of notation, in what follows we abbreviate

20 $$\begin{aligned} \xi :=\frac{\eta }{l^2}. \end{aligned}$$

Once we have computed *u*, we may recover *v* using that by (15)

21 $$\begin{aligned} \partial _t u&= l^{-2}\partial _\tau u= \frac{c}{\eta ^{-2}+\tau ^2} \frac{1}{l^4} v. \end{aligned}$$

We remark that for $\eta$ much larger than $l^{2}$ at time $\tau =\frac{1}{l^4}$ it holds that $\frac{c}{\eta ^{-2}+ l^{-4}} l^{-4}\approx \frac{c}{l^{-4}} l^{-4}=c$. We further note that here *v* is evaluated at time $\tau =\frac{1}{l^2}t$, but corresponds to the mode $(l-1,\eta )$ of the vorticity. The equation (19) has an explicit solution in terms of special functions, which we use to establish the following theorem:

### Theorem 4

Let $\xi =\frac{\eta }{l^2} \in {\mathbb {R}}$, then the problem (19)

$$\begin{aligned} \partial _t^2 u + \frac{c^2}{\xi ^{-2} + t^2} u =0 \end{aligned}$$

on $(-1,1)$ has an explicit *scattering matrix* $M=M(c,\xi )$ (see Proposition 3) such that

$$\begin{aligned} \begin{pmatrix} u(1) \\ \partial _tu(1) \end{pmatrix} = M \begin{pmatrix} u(-1) \\ \partial _tu(-1) \end{pmatrix}. \end{aligned}$$

In particular, if $0<c<\frac{1}{2}$ and $\xi \gg c^{-1}$, and we further assume that $\partial _tu(-1)\geqq 0.5 |u(-1)|$, then it follows that

22 $$\begin{aligned} \begin{pmatrix} u(1) \\ \partial _tu(1) \end{pmatrix} \approx \xi ^{\gamma } u(-1) \begin{pmatrix} \pi c^2 \\ \pi c^2 \end{pmatrix}, \end{aligned}$$

where $$\gamma =\sqrt{1-4c^2}$$.

We thus observe that the logarithmic correction seen in Section 3 here manifests in a modified exponent. As the explicit solution in terms of hypergeometric functions is technically involved but rather opaque, the proof of Theorem 4 is given in “Appendix A”.

In what follows we instead discuss the underlying mechanism by approximating $\frac{c^2}{\xi ^{-2} + t^2}$ by $\frac{c^2}{t^2}$ and $c^2 \xi ^2$ on sub-intervals of $(-1,1)$. The interactions between these regimes then yields a correction to the exponent. We remark that in this section our splitting corresponds to a more rough approximation in order to introduce ideas. In Section 5.1 we choose our splitting more carefully and consider also the influence of other modes.

Let thus $\xi \in {\mathbb {R}}$ be given and suppose that $\xi \gg c^{-1}$ is large (otherwise we may consider the system (17)). Then on the intervals $(-1,\xi ^{-1})$ and $(\xi ^{-1},1)$, it holds that

$$\begin{aligned} \frac{1}{\xi ^{-2}+t^2} \approx \frac{1}{t^2}, \end{aligned}$$

and we hence consider the second order ODEs

23 $$\begin{aligned} \partial _{t}^2 u + \frac{c^2}{t^2} u =0 \end{aligned}$$

on $$\xi ^{-1}<|t|<1$$ and

24 $$\begin{aligned} \partial _{t}^2 u + c^2 \xi ^2 u =0 \end{aligned}$$

on $$|t|<\xi ^{-1}$$.

In order to understand the mapping properties of Theorem 4 we thus consider the following scheme:

1. We prescribe initial data $(u(-1),\partial _tu(-1))$ and solve (23) to obtain $(u(-\xi ^{-1}),\partial _tu(-\xi ^{-1}))$ in Lemma 4
2. We then solve (24) to obtain $(u(\xi ^{-1}),\partial _tu(\xi ^{-1}))$ in Lemma 3
3. Finally, we solve (23) on $(\xi ^{-1},1)$ by again using Lemma 4.
4. Combining these three maps, we show in Proposition 3 that the map $(u,\partial _tu)|_{t=-1}\mapsto (u,\partial _tu)|_{t=1}$ has singular values of size $\xi ^{\gamma }$ with $\gamma =\gamma (c)<1$.

In the following Section 4.1 we then iterate this evolution in *l* to obtain sharp Gevrey 2-type norm inflation results for this model problem.

We remark that (23) is the scaling critical Schrödinger problem, which has a general solution

$$\begin{aligned} c_1 |t|^{\frac{1}{2}(1+\sqrt{1-4c^2})} + c_2 |t|^{\frac{1}{2}(1-\sqrt{1-4c^2})}, \end{aligned}$$

provided $0<c < \frac{1}{2}$. We in particular note that the exponents $\gamma _1=\frac{1}{2}+\sqrt{\frac{1}{4}-c^2}$ and $\gamma _2=\frac{1}{2}-\sqrt{\frac{1}{4}-c^2}$ are strictly between 0 and 1 and $\gamma _1+\gamma _2=1$.

### Lemma 3

Let $c \ne 0, \eta >0, k \in {\mathbb {Z}}\setminus \{0\}$, then the solution $u \in C^2$ of

25 $$\begin{aligned} \partial _{t}^2 u + c^2\xi ^{2} u =0 \end{aligned}$$

in $$(-\xi ^{-1}, \xi ^{-1})$$ satisfies

$$\begin{aligned} \begin{pmatrix} u(\xi ^{-1}) \\ \partial _tu(\xi ^{-1}) \end{pmatrix} = \begin{pmatrix} \cos (2c) &{}\quad \frac{1}{c}\xi ^{-1}\sin (2c) \\ -c \xi \sin (2c) &{}\quad \cos (2c) \end{pmatrix} \begin{pmatrix} u(-\xi ^{-1}) \\ \partial _tu(-\xi ^{-1}) \end{pmatrix}. \end{aligned}$$

In particular, we observe that for $\xi \gg c^{-2}$ the bottom left matrix entry is by far the largest, while $\cos (2c)\approx 1$.

### Proof of Lemma 3

We observe that a general solution of (25) is given by $c_1 \sin (c\xi t) + c_2 \cos (c \xi t)$. One may then verify that

$$\begin{aligned} \begin{pmatrix} u(t) \\ \partial _tu(t) \end{pmatrix} = \begin{pmatrix} \cos (c\xi (t+\xi ^{-1})) &{}\quad \frac{1}{c}\xi ^{-1}\sin (c\xi (t+\xi ^{-1})) \\ -c\xi \sin (c\xi (t+\xi ^{-1})) &{}\quad \cos (c\xi (t+\xi ^{-1})) \end{pmatrix} \begin{pmatrix} u(-\xi ^{-1}) \\ \partial _tu(-\xi ^{-1}) \end{pmatrix} \end{aligned}$$

satisfies the initial conditions. $\quad \square$

On the exterior intervals we similarly obtain an explicit solution, but with very different dependence on $\xi$.

### Lemma 4

Let $0<c<\frac{1}{2}$ and $\xi >1$ then the solution of $u \in C^2$ of

26 $$\begin{aligned} \partial _{t}^2 u + \frac{c^2}{t^2} u =0 \text { in } (-1,1)\setminus (-\xi ^{-1},\xi ^{-1}) \end{aligned}$$

satisfies

$$\begin{aligned} \begin{pmatrix} u(\xi ^{-1}) \\ \partial _tu(\xi ^{-1}) \end{pmatrix} = \begin{pmatrix} \xi ^{-\gamma _1} &{}\quad \xi ^{-\gamma _2} \\ \gamma _1\xi ^{1-\gamma _1} &{}\quad \gamma _2 \xi ^{1-\gamma _2} \end{pmatrix} \begin{pmatrix} 1 &{}\quad 1 \\ \gamma _1 &{}\quad \gamma _2 \end{pmatrix}^{-1} \begin{pmatrix} u(1) \\ \partial _tu(1) \end{pmatrix}, \end{aligned}$$

and

$$\begin{aligned} \begin{pmatrix} u(-\xi ^{-1}) \\ \partial _tu(-\xi ^{-1}) \end{pmatrix} = \begin{pmatrix} \xi ^{-\gamma _1} &{}\quad \xi ^{-\gamma _2} \\ -\gamma _1\xi ^{1-\gamma _1} &{}\quad -\gamma _2 \xi ^{1-\gamma _2} \end{pmatrix} \begin{pmatrix} 1 &{}\quad 1 \\ - \gamma _1 &{}\quad - \gamma _2 \end{pmatrix}^{-1} \begin{pmatrix} u(-1) \\ \partial _tu(-1) \end{pmatrix}, \end{aligned}$$

where $$\gamma _{1,2}=\frac{1}{2}\pm \sqrt{\frac{1}{4}-c^2}$$.

### Proof of Lemma 4

We note that a general solution of (26) is given by

$$\begin{aligned} c_{1,\pm } |t|^{\gamma _1} + c_{2,\pm } |t|^{\gamma _2}, \end{aligned}$$

where $c_{\cdot , \pm }$ are constant on each connected component of the domain. We may then again verify that

$$\begin{aligned} \begin{pmatrix} u(t) \\ \partial _tu(t) \end{pmatrix} = \begin{pmatrix} |t|^{\gamma _1} &{}\quad |t|^{\gamma _2} \\ \text {sgn}(t)\gamma _1 |t|^{\gamma _1-1} &{}\quad \text {sgn}(t)\gamma _2 |t|^{\gamma _2-1} \end{pmatrix} \begin{pmatrix} 1 &{}\quad 1 \\ \text {sgn}(t) \gamma _1 &{}\quad \text {sgn}(t) \gamma _2 \end{pmatrix}^{-1} \begin{pmatrix} u(1) \\ \partial _tu(1) \end{pmatrix} \end{aligned}$$

satisfies the boundary conditions. $\quad \square$

### Proposition 3

Let $0<c<\frac{1}{2}$ and let $u \in C^1$ be a solution of (23) and (24). Then there exists an explicitly computable matrix $M=M(c, \xi )$ such that

27 $$\begin{aligned} \begin{pmatrix} u(1) \\ \partial _tu(1) \end{pmatrix} = M \begin{pmatrix} u(-1) \\ \partial _tu(-1) \end{pmatrix}. \end{aligned}$$

In particular, if $0<c<\frac{1}{2}$ and $\xi \gg c^{-1}$, and we further assume that $|u(-1)|\geqq 2 |u'(-1)|$, then it follows that

28 $$\begin{aligned} \begin{pmatrix} u(1) \\ \partial _tu(1) \end{pmatrix} \approx \xi ^{\gamma } u(-1) \begin{pmatrix} 8c^2 \\ 8c^2 \end{pmatrix}, \end{aligned}$$

where $$\gamma =\sqrt{1-4c^2}$$.

### Proof of Proposition 3

Combining Lemma 3 and Lemma 4, we obtain that our data at $t=-1$ and $t=1$ are related by

$$\begin{aligned}&\begin{pmatrix} 1 &{}\quad 1 \\ \gamma _1 &{}\quad \gamma _2 \end{pmatrix} \begin{pmatrix} \xi ^{-\gamma _1} &{} \xi ^{-\gamma _2} \\ \gamma _1\xi ^{1-\gamma _1} &{}\quad \gamma _2 \xi ^{1-\gamma _2} \end{pmatrix}^{-1} \begin{pmatrix} \cos (2c) &{}\quad \frac{1}{c}\xi ^{-1}\sin (2c) \\ -c \xi \sin (2c) &{}\quad \cos (2c) \end{pmatrix}\\&\quad \begin{pmatrix} \xi ^{-\gamma _1} &{}\quad \xi ^{-\gamma _2} \\ -\gamma _1\xi ^{1-\gamma _1} &{}\quad -\gamma _2 \xi ^{1-\gamma _2} \end{pmatrix} \begin{pmatrix} 1 &{}\quad 1 \\ - \gamma _1 &{}\quad - \gamma _2 \end{pmatrix}^{-1}. \end{aligned}$$

We now compute

$$\begin{aligned} \begin{pmatrix} \xi ^{-\gamma _1} &{}\quad \xi ^{-\gamma _2} \\ \gamma _1\xi ^{1-\gamma _1} &{}\quad \gamma _2 \xi ^{1-\gamma _2} \end{pmatrix}^{-1}&= -\frac{1}{\gamma } \begin{pmatrix} \gamma _2 \xi ^{1-\gamma _2} &{}\quad -\xi ^{-\gamma _2} \\ -\gamma _1\xi ^{1-\gamma _1} &{}\quad \xi ^{-\gamma _1} \end{pmatrix}, \\ \begin{pmatrix} 1 &{} 1 \\ - \gamma _1 &{} - \gamma _2 \end{pmatrix}^{-1}&= \frac{1}{\gamma } \begin{pmatrix} -\gamma _2 &{}\quad -1 \\ \gamma _1 &{}\quad 1 \end{pmatrix}, \end{aligned}$$

where we used $\gamma _1+\gamma _2=1$ and computed the determinants as

$$\begin{aligned} \gamma _1-\gamma _2=:\gamma =\sqrt{1-4c^2}\approx 1. \end{aligned}$$

Since $\xi \gg 1$, $\gamma _1=\frac{1}{2}+\sqrt{\frac{1}{4}-c^2} \approx 1-c^2/2\approx 1$, $\gamma _2= \frac{1}{2}-\sqrt{\frac{1}{4}-c^2}\approx c^2\ll 1$ it follows that the largest powers of $\xi$ are dominant. We may thus approximate:

$$\begin{aligned} \begin{pmatrix} u(-\xi ^{-1}) \\ \partial _tu(-\xi ^{-1}) \end{pmatrix}&=\frac{1}{\gamma } \begin{pmatrix} \xi ^{-\gamma _1}&{}\quad \xi ^{-\gamma _2} \\ -\gamma _1\xi ^{1-\gamma _1} &{}\quad -\gamma _2 \xi ^{1-\gamma _2} \end{pmatrix} \begin{pmatrix} -\gamma _2 &{}\quad -1 \\ \gamma _1 &{}\quad 1 \end{pmatrix} \begin{pmatrix} u(-1) \\ u'(-1) \end{pmatrix} \\&\approx \begin{pmatrix} \xi ^{-\gamma _1}&{}\quad \xi ^{-\gamma _2} \\ -\gamma _1\xi ^{1-\gamma _1} &{}\quad -\gamma _2 \xi ^{1-\gamma _2} \end{pmatrix} \begin{pmatrix} -\gamma _2 u(-1)-\partial _tu(-1) \\ u(-1)+\partial _tu(-1) \end{pmatrix}\\&\approx u(-1) \begin{pmatrix} \xi ^{-\gamma _2} \\ -\gamma _2\xi ^{1-\gamma _2} \end{pmatrix}. \end{aligned}$$

Next, it follows that

$$\begin{aligned} \begin{pmatrix} u(\xi ^{-1}) \\ \partial _tu(\xi ^{-1}) \end{pmatrix}&= \begin{pmatrix} \cos (2c) &{}\quad \frac{1}{c}\xi ^{-1}\sin (2c)\\ -c \xi \sin (2c)&{}\quad \cos (2c) \end{pmatrix} \begin{pmatrix} u(-\xi ^{-1}) \\ \partial _tu(-\xi ^{-1}) \end{pmatrix} \\&\approx u(-1) \begin{pmatrix} \xi ^{-\gamma _2} \\ -(2c^2+\gamma _2)\xi ^{1-\gamma _2} \end{pmatrix}, \end{aligned}$$

where we omitted $\gamma _2 \frac{\sin (2c)}{c}={\mathcal {O}}(c^2)$ as a small perturbation to 1 and approximated $\sin (2c)\approx 2c$. Finally, it follows that

$$\begin{aligned} \begin{pmatrix} u(1) \\ \partial _tu(1) \end{pmatrix}&= -\frac{1}{\gamma } \begin{pmatrix} 1 &{}\quad 1 \\ \gamma _1 &{}\quad \gamma _2 \end{pmatrix} \begin{pmatrix} \gamma _2 \xi ^{1-\gamma _2} &{}\quad -\xi ^{-\gamma _2} \\ -\gamma _1\xi ^{1-\gamma _1} &{}\quad \xi ^{-\gamma _1} \end{pmatrix} \begin{pmatrix} u(\xi ^{-1}) \\ u'(\xi ^{-1}) \end{pmatrix} \\&\approx - u(-1) \begin{pmatrix} 1 &{}\quad 1 \\ \gamma _1 &{}\quad \gamma _2 \end{pmatrix} \begin{pmatrix} (2\gamma ^2+2c^2) \xi ^{1-2\gamma _2} \\ -1 \xi ^{0} \end{pmatrix}\\&\approx u(-1) \xi ^{1-2\gamma _2} \begin{pmatrix} 8c^2 \\ 8c^2 \end{pmatrix}. \end{aligned}$$

$$\square $$

We thus see that, while the evolution on the small interval $(-\xi ^{-1},\xi ^{-1})$ yields a singular value of size $\xi ^{1}$, the conjugation with the power law evolution on $|t|>\xi ^{-1}$ yields a much smaller singular value $\xi ^{\gamma }\ll \xi ^{1}$.

Having establish a precise description of the evolution for times close to a single resonant time $\tau \approx \frac{1}{l}$, in the following we consider an iterated model to study the norm inflation in Gevrey spaces and the associated asymptotic behavior.

### Model Echo Chains and Modified Exponents

In Section 3 we have studied chains of echoes for the linear problem (4) and have established norm inflation with a factor $\exp (C \sqrt{\eta })$. However, in that case our proof limited us to considering only $\eta$ such that $c \ln (1+\eta ^2)$ is not too large.

In what follows we instead consider an iterated version of the model of Section 4, which does not possess such an obstruction. In particular, combining the behavior of infinitely many modes $\eta _j$ with $\eta _j\rightarrow \infty$ we construct solutions which exhibit norm inflation for arbitrarily large times and do not converge as time tends to infinity. However, despite the failure of the convergence of the vorticity, the velocity field is shown to converge.

We briefly recall the approximations made in the preceding sections of this article. We started with the 2D Euler equations close to Couette flow

$$\begin{aligned} \partial _t\omega + y\partial _x \omega + v \cdot \nabla \omega =0, \end{aligned}$$

and focused on perturbations of the form $\omega = c \cos (x+ty)+ \varepsilon \omega _*$. Omitting the transport by $c\frac{\sin (x+ty)}{1+t^2}$ and changing to variables $(x+ty,y)$, we obtain

$$\begin{aligned} \partial _t\omega + c\sin (x) \partial _y \varDelta _t^{-1} \omega + \varepsilon \nabla ^\bot \varDelta _t^{-1} \omega \cdot \nabla \omega =0. \end{aligned}$$

In particular, we note that this equation formally conserves $\Vert c \cos (x)+ \varepsilon \omega \Vert _{L^2}$. Omitting the nonlinearity by letting $\varepsilon \downarrow 0$, we lose this conserved quantity, but obtain an explicit Fourier problem with nearest-neighbor-interactions:

29 $$\begin{aligned} \begin{aligned}&\partial _t{\tilde{\omega }}(t,k,\eta ) + c \frac{\eta }{(k-1)^2+(\eta -(k-1)t)^2} {\tilde{\omega }}(t,k-1,\eta ) \\&\quad - c \frac{\eta }{(k+1)^2+(\eta -(k+1)t)^2} {\tilde{\omega }}(t,k+1,\eta )=0. \end{aligned} \end{aligned}$$

Here with slight abuse of notation we replaced *c* by *c*/2 for brevity. This further highlights resonant times, where $\eta -(k\pm 1)t\approx 0 \Leftrightarrow t \approx \frac{\eta }{k \pm 1}$. After studying the system (29) in Sections 2 and 3, in Section 4 we further introduced a model problem (15) that focuses solely on resonant modes *v* ($(l,\eta )$ such that $\eta -lt \approx 0$) and their neighbors u:

30 $$\begin{aligned} \begin{aligned}&\partial _\tau u - \frac{c}{\eta ^{-2}+\tau ^2} \frac{1}{l^2} v(\tau ) =0, \\&\partial _\tau v + \frac{c}{\eta ^{-2}+l^{-4}} \frac{1}{l^2} u(\tau ) =0. \end{aligned} \end{aligned}$$

In particular, we showed that $v|_{\tau =\tau _1}$ is approximately of size $c (\frac{\eta }{l^2})^\gamma u|_{\tau =\tau _0}$.

Building on the single resonance results of Theorem 4, we construct the following iteration scheme:

- Let $k \in {\mathbb {N}}$, $k>1$ and $\eta \in {\mathbb {R}}$ be given and define $\tau _k=\frac{1}{2}(\frac{1}{k-1}+\frac{1}{k})$. We then prescribe $(u,\partial _\tau u)|_{\tau _k}$ and use equation (19) to determine $(u,\partial _t u)|_{\tau _{k-1}}$.
- Relabeling *v* of the previous step as *u* of the case $k-1$ and using (21) we prescribe
  $$\begin{aligned} \begin{pmatrix} u_{k-1} \\ \partial _tu_{k-1} \end{pmatrix}|_{\tau =\tau _{k-1}} := \begin{pmatrix} c^{-1} \ \partial _tu_k \\ c u_k \end{pmatrix}|_{\tau =\tau _{k-1}}. \end{aligned}$$
  We then again use equation (19) to determine $(u,\partial _tu)|_{\tau _{k-2}}$.
- We iterate this procedure until we reach $\tau _1$, where we define $\tau _0=1.5$.

Recalling the construction of the model problem of Section 4, $(u,\partial _t u)_{\tau =\tau _k}$ corresponds to prescribing $({\tilde{\omega }}(t,k, \eta ), {\tilde{\omega }}(t,k-1,\eta ))$ at time $t=\frac{1}{2}(\frac{\eta }{k-1}+\frac{\eta }{k})$ and $(u,u')|_{\tau =\tau _0}$ corresponds to the value of the modes $(1,\eta )$ and $(0,\eta )$ at time $t=1.5 \eta$.

#### Theorem 5

Let $k \in {\mathbb {N}}$ and $\eta \in {\mathbb {R}}$ be given and prescribe $(u,\partial _t u)|_{\tau =\tau _k}=(1,0)$. Then, the above iteration scheme yields that

$$\begin{aligned} (u, \partial _t u) |_{\tau =\tau _0} \approx (c, c^{2})c^{k-1} \left( \frac{\eta ^k}{(k!)^2}\right) ^\gamma , \end{aligned}$$

where $\gamma =\sqrt{1-4c^2}\ne 1$. In particular, choosing *k* maximally for $c,\eta$ fixed, we obtain a growth factor

$$\begin{aligned} \max _{k} c^{k}\left( \frac{\eta ^k}{(k!)^2}\right) ^\gamma \sim e^{2\gamma \sqrt{c^{1/\gamma }\eta }} \end{aligned}$$

consistent with a Gevrey regularity class.

#### Proof

Using the result of Theorem 4 (specifically equation (22)), we observe that

$$\begin{aligned} \begin{pmatrix} u_{k-1} \\ \partial _tu_{k-1} \end{pmatrix}|_{\tau =\tau _{k-1}} \approx \partial _tu_k(\tau _{k})\xi ^{\gamma } \begin{pmatrix} 8c \\ 8c^3 \end{pmatrix}. \end{aligned}$$

In particular, we note that $u_{k-1}\approx c^{-2}\partial _tu_{k-1}$ and we may thus apply Theorem 4 again. We repeat this process another $k-1$ times, where $\xi =\frac{\eta }{k^2}$ changes in each step, and thus obtain the claimed growth factor

$$\begin{aligned} c^k \left( \frac{\eta ^k}{(k!)^2}\right) ^{\gamma }. \end{aligned}$$

Considering $c\eta ^{\gamma }$ large and *k* large, by Stirling’s approximation it holds that

$$\begin{aligned} c^{k} \left( \frac{\eta ^{k}}{(k!)^2} \right) ^{\gamma } \sim c^{k} \left( \eta ^{k} \frac{e^{2k}}{2\pi k k^{2k}} \right) ^{\gamma }. \end{aligned}$$

Choosing *k* as approximately $\sqrt{c^{\frac{1}{\gamma }}\eta }$ (rounding up or down), we obtain a cancellation of $c^{k }\left( \frac{\eta ^{k}}{k^{2k}}\right) ^{\gamma }=1$ and thus

$$\begin{aligned} e^{2\gamma \sqrt{c^{1/\gamma }\eta }} \left( \frac{1}{2\pi \sqrt{c^{1/\gamma }\eta }} \right) ^\gamma \end{aligned}$$

as the maximal growth factor. $\quad \square$

As a corollary, for this model we can construct initial data in a critical Gevrey regularity class.

#### Theorem 6

Consider the chained two mode model with $0<c<\frac{1}{2}$ and $\gamma =\sqrt{1-4c^2}$. Then there exists $s=s(\gamma )$ and $C>0$ such that for every $\varepsilon >0$ and every $\sigma _0 \in {\mathbb {R}}$, there exists initial data $u_0 \in {\mathcal {G}}_{C, \frac{1}{s}}$ (see Section 1.1.1 for a definition) such that

31 $$\begin{aligned} \Vert u_0\Vert _{{\mathcal {G}}_{C, \frac{1}{s}}}< \varepsilon , \end{aligned}$$

and such that for every ${\tilde{C}}>0$,

$$\begin{aligned} \lim _{t \rightarrow \infty } \Vert u(t)\Vert _{{\mathcal {G}}_{s,{\tilde{C}}}}=\infty . \end{aligned}$$

Furthermore, *u*(*t*) does converge in $H^{\sigma }$, $\sigma <\sigma _0$, but diverges in $H^{\sigma }, \sigma >\sigma _0$.

In particular, choosing $-1< \sigma _0 <0$, we find initial data, arbitrarily small in the critical Gevrey regularity class, such that the vorticity does not converge in $L^2$ as $t \rightarrow \infty$, but the velocity field does converge.

#### Proof

For any given $\eta$, let $k_\eta$ be the associated maximizer of the growth factor obtained in Theorem 5 and let $g(\eta )$ denote that growth factor.

Let now $\psi \in \cap _{\sigma <\sigma _0}H^{\sigma }({\mathbb {R}})$ be given and prescribe as initial data

$$\begin{aligned} u= \int _{\eta } \frac{1}{g(\eta )} {\hat{\psi }}(\eta ) e^{i \eta y + i k_{\eta }x}. \end{aligned}$$

Since $g(\eta )\approx \exp (C \sqrt{\eta })$ for $C=C(c)$, this function is in a Gevrey class.

Then by construction, the $k=1$ mode will asymptotically be given by

$$\begin{aligned} \int _{\eta } g(\eta ) \frac{1}{g(\eta )} {\hat{\psi }}(\eta ) e^{i\eta y + i x}= \psi (y) e^{ix} \end{aligned}$$

$$\square $$

Building on the insights obtained in this model problem in the next Section we consider the full problem.

## Echo Chains as a Linear Mechanism and Modified Scattering

In the previous section we have shown that for large $\eta$ a linear growth factor $\frac{\eta }{l^2}$ cannot be expected to be accurate anymore. Indeed, the logarithmic corrections in the Duhamel iteration are much larger than the prior “leading term”. Instead we expect to see a modified exponent, which is less than 1 due to cancellations with neighboring modes. In what follows we consider the full model (7)

32 $$\begin{aligned} \begin{aligned}&\partial _\tau \omega (\tau ,k,\eta ) + c \frac{1}{(k-1)^2} \frac{1}{\eta ^{-2} +(\frac{1}{k-1}-\tau )^2} \omega (\tau ,k-1,\eta ) \\&\quad - c \frac{1}{(k+1)^2} \frac{1}{\eta ^{-2} +(\frac{1}{k+1}-\tau )^2} \omega (\tau ,k+1,\eta )=0 \end{aligned} \end{aligned}$$

on a time-interval around a single resonance $\frac{1}{k_0}$,

$$\begin{aligned} \left( \frac{\frac{1}{k_0+1} + \frac{1}{k_0}}{2}, \frac{\frac{1}{k_0}+\frac{1}{k_0-1}}{2}\right) . \end{aligned}$$

The main aim of this section, which is achieved in Theorem  8 of Section 5.2, is then to show that the evolution of this full model is largely determined by the evolution of the three modes $k_0+1, k_0, k_0-1$. To this end, in Section 5.1 we first introduce a model problem focusing on just these three modes, which should be seen as a refinement of the two mode model of Section 4. Here, as in Section 4 a key challenge lies in capturing the asymptotic dependence on $\xi =\frac{\eta }{k^2}$ for $\xi$ very large. Subsequently, we employ a bootstrap approach in the (sub)subsections of Section 5.2 to show that this three mode model indeed captures the full growth mechanisms and that all other modes can be controlled. Taking these results as building blocks, in Section 5.3 we combine the resonances to construct echo chains which exhibit Gevrey 2 growth and furthermore combine countably many echo chains to construct solutions with critical stability/blow-up behavior.

Considering that

$$\begin{aligned} \frac{1}{k_0\pm 1}- \frac{1}{k_0}= \frac{1}{k_0(k_0\pm 1)} \leqq \frac{2}{k_0^2}, \end{aligned}$$

and the central role of $\tau =\frac{1}{k_0}$, compared to Section 4 we again change variables as

33 $$\begin{aligned} t= k_0^{2}\left( \tau -\frac{1}{k_0}\right) \in \left( -\frac{k_0}{2(k_0+1)},\frac{k_0}{2(k_0-1)}\right) =:(t_0,t_1). \end{aligned}$$

Then $\partial _\tau = k_0^2 \partial _t$ and hence (7) reads as

34 $$\begin{aligned} \partial _t\omega (t,k,\eta ) + a(k-1) \omega (t,k-1,\eta )- a(k+1) \omega (t,k+1,\eta )=0, \end{aligned}$$

where

35 $$\begin{aligned} a(k_0)= \frac{c}{\left( \frac{\eta }{k_0^2}\right) ^{-2}+t^2}, \end{aligned}$$

and we abbreviate

36 $$\begin{aligned} \xi := \frac{\eta }{k_0^2}, \end{aligned}$$

and for $$k\ne k_0$$,

37 $$\begin{aligned} a(k)&= \frac{1}{k^2k_0^2} \frac{c}{\eta ^{-2} + (\frac{1}{k}-\frac{1}{k_0}-k_0^{-2}t)^2} \end{aligned}$$

38 $$\begin{aligned}&= \frac{c}{\eta ^{-2}k_0^2 k^2 + (k_0-k -\frac{k}{k_0}t)^2}. \end{aligned}$$

In particular, we note that since we only consider the resonant interval around $k_0$ and are thus far from other resonant times $|a(k)|\leqq 4 c$ for any $k\ne k_0$. In contrast, $a(k_0)$ at time $t=0$ is of size $c \xi ^{2} \gg 1$.

We remark that in Section 4 we made several simplifications compared to the full model:

1. We approximated $a(k_0\pm 1)\approx \pm c$, which allowed us to compute explicit solutions. In the following we need to show that this is a valid approximation, that is the evolution of the full problem can be estimated above and below by the approximate evolution.
2. In Section 4 we considered a two-mode model involving just $k_0$ and $k_0-1$. Instead we show that the precise behavior is more accurately captured by the three-mode model involving $k_0-1,k_0,k_0+1$, which yields a change of the exponent $\gamma$ (involving $2c^2$ in place of $c^2$).
3. In view of the sizes of *a*(*k*) in the following Section 5.1 we at first again neglect all except the three modes $k_0-1,k_0,k_0+1$. We call this the *three-mode model*. In contrast to the two-mode model of Section 4 we here do not approximate the coefficient functions. In Section 5.2 we then discuss the full problem incorporating all modes and prove that indeed all other modes can treated as perturbations in a bootstrap approach.

### The Three-Mode Model

In this section we introduce the solution operator of the homogeneous three-mode model:

39 $$\begin{aligned} \partial _tu(k)&+ a(k+1)u(k+1)-a(k-1)u(k-1)=0, \end{aligned}$$

40 $$\begin{aligned} a(k)&= {\left\{ \begin{array}{ll} a(k_0\pm 1)= \frac{c}{\xi ^{-2}(\frac{k_0\pm 1}{k_0})^2 + (1\pm \frac{k_0\pm 1}{k_0}t)^2}, \\ a(k_0)= \frac{c}{\xi ^{-2}+t^2},\\ 0. \end{array}\right. } \end{aligned}$$

That is, we consider only the modes $k_0-1, k_0, k_0+1$ and neglect all other modes as “inhomogeneities”. This allows for a clearer discussion of the growth and decay mechanisms and serves to introduce the techniques of proof used in the different regimes. It is also an important preliminary step before studying the behavior of the full problem in Theorem 8 of Section 5.2. More precisely, we will (with considerable technical effort) consider the full problem as a perturbation by an inhomogeneity, which we control by means of a bootstrap approach.

We recall that, by the results of Section 4, the heuristic of the approximate model $a(k_0\pm 1)\approx c$ suggests a power law behavior of solutions. As for the present case of exact coefficients an explicit solution is not feasible anymore, so we establish a comparison estimate. We argue in multiple steps:

- By symmetry it holds that $u(k_0+1)+u(k_0-1)=\text {const.}$. We hence to some extent reduce to a two-mode model. However, as $a(k_0+1)\ne a(k_0-1)$ in this model the problem does not completely decouple.
- We first establish a rough power law upper bound on the growth of solutions on $(-t_0,-t)$ as $t \downarrow 0$. This is achieved by concatenating multiple small time estimates.
- Subsequently, we iteratively improve this bound to $t^{\gamma _2-1}$, $\gamma _2= \frac{1}{2}-\sqrt{\frac{1}{4}-2c^2}$ upper and lower bounds similar as in Section 4 (here in the formula for $\gamma _2$ the $c^2$ is replaced by $2c^2$ due to the third mode). This step relies on reformulations of the ODE system as second order ODEs and integrating these.
- We then show that the resonant mode is decreasing as $t^{\gamma _2}$ while the neighboring modes grow like $t^{\gamma _2-1}$both with an upper and lower bound.
- Combining these results, we construct solution operators on the intervals $I_1=(t_0,-\frac{d}{\xi })$, $I_3=(\frac{d}{\xi },t_1)$ with $d=c^{-1}$. On the resonant interval $I_2=(-\frac{d}{\xi }, \frac{d}{\xi })$ we instead use a Duhamel iteration argument to construct the solution operator.
- Concatenating the solution operators we obtain the solution operator from time $t_0$ to $t_1$ and show that it exhibits analogous $(\frac{\eta }{k_0^2})^{\gamma }$ growth behavior to the model problem of Section 4. In Section 5.2 we then show that this behavior persists in the full problem.

The main results of this section are summarized in the following theorem:

#### Theorem 7

(The full solution, homogeneous case) Let $\xi =\frac{\eta }{k_0^2} \gg 1$ and $0<c< 0.2$ be given and let $t_0=-\frac{1}{2}\frac{k_0}{k_0+1}$ and $t_1=\frac{1}{2}\frac{k_0}{k_0-1}$. We consider the ODE system (39) for $u_1= \frac{u(k_0+1)-u(k_0-1)}{2}, u_2=u(k_0), u_3=\frac{u(k_0+1)+u(k_0-1)}{2}:$

41 $$\begin{aligned} \partial _tu + M(t) u =0, \end{aligned}$$

where

$$\begin{aligned} M(t)= \begin{pmatrix} 0 &{}\quad a(k_0) &{}\quad a(k_0+1)-a(k_0-1)\\ -(a(k_0+1)-a(k_0-1)) &{}\quad 0 &{}\quad -a(k_0+1)+a(k_0-1) \\ 0 &{}\quad 0 &{}\quad 0 \end{pmatrix} \end{aligned}$$

on the interval $(t_0,t_1)$.

Suppose that at time $t_0=-\frac{1}{2}\frac{k_0}{k_0+1}$ it holds that $u(k_0) \geqq 0.5 \max (u)$. Then at time $t_1=\frac{1}{2}\frac{k_0}{k_0-1}$ it holds that

$$\begin{aligned} u_1(t_1)&\approx c^{2-2\gamma _2} \xi ^{\gamma } u_2(t_0),\\ u_2(t_1)&\approx c^{4-2\gamma _2}\xi ^{\gamma } u_2(t_0),\\ u_3(t_1)&= u_3(t_0), \end{aligned}$$

where $$\gamma _2=\frac{1}{2}-\sqrt{\frac{1}{4}-2c^2}$$ and $$\gamma =1-2 \gamma _2=\sqrt{1-4c^2}$$.

We note, in particular, that the exponent here is different from 1 (which was not visible in prior works due to the logarithmic constraints) and that at time $t_1$ our solution *u* satisfies the assumptions of this theorem with $k_0$ replaced by $k_0-1$. Thus, we may iteratively apply this theorem until we reach the frequency 1 and obtain the following corollary (in Section 5.3 we return to this echo chain behavior in the context of the full problem and also discuss (modified) asymptotic behavior as $t\rightarrow \infty$):

#### Corollary 1

(Echo chain, homogeneous case) Let $\eta \gg 1$ and $k_0\ll \eta$ be given. We then consider the iterated system where initially set $\xi =\frac{\eta }{k_0^2}$ and solve (41) with

$$\begin{aligned} u(t_0)= \begin{pmatrix} 0 \\ 1 \\ 0 \end{pmatrix} \end{aligned}$$

In the next step, we then decrease $k_0 \mapsto k_0-1$ and set $\xi =\frac{\eta }{(k_0-1)^2}$ and again solve (41) with new initial data being given by

$$\begin{aligned} \begin{pmatrix} u_{1}(t_0)\\ u_{2}(t_0)\\ u_{3}(t_0) \end{pmatrix}_{new} = \begin{pmatrix} 0\\ u_{1}(t_1)\\ 0 \end{pmatrix}_{old} \end{aligned}$$

We iterate this procedure another $k_0-2$ times until we have reached $k=1$. At this time it then holds that

$$\begin{aligned} u_1(1)\approx (c^{2-2\gamma _2})^{k_0} \left( \frac{\eta ^{k_0}}{(k_0!)^2} \right) ^{\gamma }. \end{aligned}$$

In particular, choosing $k_0$ maximally for given $\eta$ we obtain that $u_1(1)\approx C_1 \exp (C_2 \sqrt{\eta })$ attains a Gevrey 2 norm inflation factor.

We remark that the precise choice of $u_1,u_3$ in the update step here does not change the result as long as $u_2(t_0)$ is (comparable to) the largest entry and thus Theorem 7 can be applied.

#### Proof of Corollary 1

Let $k_0$ be given and let $t_{0}[k_0]=\frac{1}{2}(\frac{1}{k_0+1}-\frac{1}{k_0})$, $t_1[k_0]=\frac{1}{2}(\frac{1}{k_0-1}-\frac{1}{k_0})$. Then at time $t_0[k_0]$ it holds that $u_2 \geqq 0.5 \max (u)$ and we may thus apply Theorem 7 to conclude that at time $t_1[k_0]$,

$$\begin{aligned} u_1(t_1[k_0]) \approx c^{2-2\gamma _2} \xi ^{\gamma } u_2(t_0[k_0]) \geqq 0.5 \max (u(t_1[k_0])). \end{aligned}$$

We then decrease $k_0$ by 1 and obtain that, at the new initial time $t_0[k_0-1]=t_1[k_0]$, by relabeling the above,

$$\begin{aligned} u_2(t_0[k_0-1])= u_1(t_1[k_0]) \geqq 0.5 \max (u) \end{aligned}$$

again satisfies the assumptions of Theorem 7. Iterating this procedure until we reach $k_0=1$ then yields the result. $\quad \square$

Our proof proceeds by considering the three intervals $I_1=(t_0, -\frac{d}{\xi })$, $I_2=(-\frac{d}{\xi }, \frac{d}{\xi })$, $I_3=(\frac{d}{\xi }, t_1)$, where $d=c^{-1}$. The right boundary datum of each interval then serves as the left boundary datum of the next. We thus follow a similar strategy as in the proof of Theorem 4. In the Section 5.1.1 we derive upper and lower bounds for the evolution on the intervals $I_1$ and $I_3$, which we expect to be similar to the ones derived for the two mode model in Lemma 4. That is, we expect to derive asymptotics in terms of $\xi ^{-\gamma _1}, \xi ^{-\gamma _2}$ and $\xi ^{1-\gamma _1}, \xi ^{1-\gamma _2}$. In Section 5.1.2 we study the evolution on the inner interval $I_2$, where we expect an evolution similar to Lemma 4, and thus to encounter powers $\xi ^{+1}$ and $\xi ^{-1}$. Combining these asymptotics as in Proposition 3, we then obtain a combined exponent $\gamma$, which concludes our proof of Theorem 7.

#### The Interval $I_1$

As a first step in our proof of Theorem 7 we consider the evolution of the evolution of the homogeneous problem (39) on the interval

$$\begin{aligned} I_1=\left( t_0,-\frac{d}{\xi }\right) . \end{aligned}$$

Similarly to the results of Lemma 3 of Section 4 we here aim to show that for given initial data $(u_1,u_2, u_3)|_{t=t_0}$ at the right end of the interval $u_1$ has grown and $u_2$ has decreased by an appropriate power of $\xi$. Our results are summarized in the following proposition:

##### Proposition 4

(Left interval, homogeneous case) Let $\xi =\frac{\eta }{k} \gg 1$ and $0<c <0.2$ be given.

We consider the problem (39) on the interval $I_1=(t_0,-\frac{d}{\xi })$ with $d=c^{-1}$. Then the unique solution *u*(*t*) satisfies

$$\begin{aligned} u_3(t)&=u_3(t_0). \end{aligned}$$

Furthermore, it holds that

$$\begin{aligned} |u_1(t)|&\leqq C t^{\gamma _2-1} \max (u(t_0)), \\ |u_2(t)|&\leqq C t^{\gamma _2} \max (u(t_0)). \end{aligned}$$

where $$\gamma _1=\frac{1}{2}+\sqrt{\frac{1}{4}-2c^2}$$, $$\gamma _2=\frac{1}{2}-\sqrt{\frac{1}{4}-2c^2}$$.

If in addition $|u_2(t_0)| \geqq 4c |u(t_0)|$, then at time $t=-\frac{d}{\xi }$ it holds that

$$\begin{aligned} u_1\left( -\frac{d}{\xi }\right)&\approx \left( \frac{d}{\xi }\right) ^{\gamma _2-1}\frac{c}{\gamma _2-\gamma _1} u_2(t_0), \\ u_2\left( -\frac{d}{\xi }\right)&\approx \left( \frac{d}{\xi }\right) ^{\gamma _2} \frac{\gamma _1}{\gamma _2-\gamma _1}u_2(t_0). \end{aligned}$$

For later reference we note that $u_1(-\frac{d}{\xi })\gg u_2(-\frac{d}{\xi })$ and that $u_1(-\frac{d}{\xi })(\frac{d}{\xi })\approx c \ u_2(-\frac{d}{\xi })$.

We may write the homogeneous three-mode model (39) as

42 $$\begin{aligned} \partial _t\begin{pmatrix} u(k_0-1) \\ u (k_0) \\ u(k_0+1) \end{pmatrix} + \begin{pmatrix} 0 &{}\quad a &{}\quad 0 \\ - b_1 &{}\quad 0 &{}\quad b_2 \\ 0 &{}\quad -a &{}\quad 0 \end{pmatrix} \begin{pmatrix} u(k_0-1) \\ u (k_0) \\ u(k_0+1) \end{pmatrix} =0, \end{aligned}$$

where $a=\frac{c}{\xi ^{-2}+t^2}$, $b_1\ne b_2\approx c$. For this structure it is apparent that $u_3:=\frac{1}{2}(u(k_0-1)+u(k_0+1))$ is conserved. However, compared to the two mode model studied in Lemma 3 here $u_3$ serves as an inhomogeneity for the remaining modes, as shown in the following lemma. This inhomogeneity then leads to a particular solution constructed in Lemma 7. Furthermore, unlike in the model setting of Section 4 the resulting problem does not allow for explicit solutions. Instead we show by successive improvement in Lemma 6 that the solutions can be bounded by an appropriate power law.

##### Lemma 5

(Reduction 1) Consider the problem (42), then it holds that

$$\begin{aligned} u(k-1) + u(k+1) =\text {const.}. \end{aligned}$$

We hence introduce the change of unknowns and notation

$$\begin{aligned} u_1&= \frac{1}{2}(u(k_0-1)-u(k_0+1)), \\ u_2&= u(k_0), \\ u_3&= \frac{1}{2}(u(k_0-1)+u(k_0+1)), \end{aligned}$$

where $u_3$ is invariant under the evolution and $(u_1,u_2)$ solve

43 $$\begin{aligned} \partial _t\begin{pmatrix} u_1 \\ u_2 \end{pmatrix} + \begin{pmatrix} 0 &{}\quad a \\ -b &{}\quad 0 \end{pmatrix} \begin{pmatrix} u_1 \\ u_2 \end{pmatrix} = \begin{pmatrix} (b_1+b_2)u_3 \\ (b_1-b_2)u_3. \end{pmatrix} , \end{aligned}$$

where $$b=b_1+b_2$$.

##### Proof of Lemma 5

We observe that

$$\begin{aligned} \partial _t(u(k_0+1)+u(k_0-1))= -a u(k_0)+ a u(k_0)=0 \end{aligned}$$

and hence $u_3$ is conserved. The equation satisfied by $u_1,u_2$ is then just a reformulation of equation (42). $\quad \square$

In order to solve (43) we first focus on the special case $u_3=0$,

44 $$\begin{aligned} \partial _t\begin{pmatrix} u_1 \\ u_2 \end{pmatrix} + \begin{pmatrix} 0 &{}\quad a \\ -b &{}\quad 0 \end{pmatrix} \begin{pmatrix} u_1 \\ u_2 \end{pmatrix} =0. \end{aligned}$$

That is, we study the homogeneous solutions of (43). In Lemma 7 we then construct a particular solution for the case $u_3\ne 0$.

As a first heuristic, note that on $I_1=(t_0,-\frac{d}{\xi })$ it seems reasonable to approximate

45 $$\begin{aligned} a(t)&= \frac{c}{\xi ^{-2}+t^2} \approx \frac{c}{t^2}, \end{aligned}$$

46 $$\begin{aligned} b(t)&\approx 2c. \end{aligned}$$

This approximated problem can be explicitly solved and suggests that

47 $$\begin{aligned} \begin{pmatrix} u_1 \\ u_2 \end{pmatrix} \approx \begin{pmatrix} \frac{\gamma _1}{c} |t|^{\gamma _1-1} &{} \frac{\gamma _2}{c} |t|^{\gamma _2-1} \\ |t|^{\gamma _1} &{} |t|^{\gamma _2} \end{pmatrix} \begin{pmatrix} \alpha \\ \beta \end{pmatrix}, \end{aligned}$$

for suitable constants $\alpha , \beta$ and $\gamma _1=\frac{1}{2}+ \frac{1}{2}\sqrt{1-8c^2}$, $\gamma _2=\frac{1}{2}+ \frac{1}{2}\sqrt{1-8c^2}$. In the following we will show that this heuristic is indeed valid in the sense that the actual solution operator has the same asymptotic power law behavior as |*t*| becomes small.

In order to establish these asymptotics we make use of a self-improving estimate. That is we will assume for the moment that there exists some $\sigma <\infty$ such that

48 $$\begin{aligned} |u_1(t)| \leqq C |t|^{-\sigma } |u(t_0)|, \end{aligned}$$

and show that then $u_1$ and $u_2$ necessarily also satisfy the upper lower bounds given in Proposition 4. This a priori assumption is established at the end of this Section in Lemma 8.

##### Lemma 6

(Improvement) Consider the problem (44) on the interval $I_1=(t_0,-\frac{d}{\xi })$ and suppose that $u_1$ satisfies the upper bound (48).

Then there exists a constant $C>0$ (independent of $\xi , \varepsilon$, possibly dependent on *c*), such that

49 $$\begin{aligned} |u_1(t)|&\leqq C |t|^{\gamma _2-1} |u(t_0)|, \end{aligned}$$

50 $$\begin{aligned} |u_2(t)|&\leqq C |t|^{\gamma _2} |u(t_0)|, \end{aligned}$$

where $$\gamma _2= \frac{1}{2}-\frac{1}{2}\sqrt{1-8c^2}$$.

Furthermore, if $|u_2(t_0)|\geqq 0.5 |u(t_0)|$, then at time $t=-\frac{d}{\xi }$ it holds that

51 $$\begin{aligned} \begin{aligned} |u_1(t)|&\geqq C/2 \left( \frac{d}{\xi }\right) ^{\gamma _2-1}|u_2(t_0)|, \\ |u_2(t)|&\leqq C/2 \left( \frac{d}{\xi }\right) ^{\gamma _2}|u_2(t_0)|. \end{aligned} \end{aligned}$$

An analogous result holds on the interval $I_3=(\frac{d}{\xi },t_1)$.

##### Proof of Lemma 6

We note that $|b(t)-2c| \leqq 10 ct$ by Taylor’s formula and hence treat it as a perturbation.

*A toy model* In order to introduce our method of proof let us first consider the approximated problem where we replace *a*(*t*) by exactly $\frac{c}{t^2}$. Then our problem can be written as

$$\begin{aligned} \partial _tu + \begin{pmatrix} 0 &{}\quad \frac{c}{t^2} \\ -2c &{}\quad 0 \end{pmatrix} u = \begin{pmatrix} 0 \\ {\mathcal {O}}(c|t|) u_1 \end{pmatrix}, \end{aligned}$$

where by assumption (48)it holds that ${\mathcal {O}}(c|t|)u_1= c{\mathcal {O}}(|t|^{1-\sigma })$. We then consider a variation of constants ansatz:

$$\begin{aligned} u(t)= \begin{pmatrix} \frac{\gamma _1}{c}|t|^{\gamma _1-1} &{}\quad \frac{\gamma _2}{c}|t|^{\gamma _2-1}\\ |t|^{\gamma _1} &{}\quad |t|^{\gamma _2} \end{pmatrix} \begin{pmatrix} \alpha \\ \beta \end{pmatrix}. \end{aligned}$$

Plugging this in, we get that

$$\begin{aligned} \begin{pmatrix} \frac{\gamma _1}{c}|t|^{\gamma _1-1} &{}\quad \frac{\gamma _2}{c}|t|^{\gamma _2-1}\\ |t|^{\gamma _1} &{}\quad |t|^{\gamma _2} \end{pmatrix} \partial _t\begin{pmatrix} \alpha \\ \beta \end{pmatrix} = \begin{pmatrix} 0 \\ {\mathcal {O}}(ct) u_1(t) \end{pmatrix}\\ \Leftrightarrow \partial _t\begin{pmatrix} \alpha \\ \beta \end{pmatrix} = \frac{c}{\gamma } \begin{pmatrix} |t|^{\gamma _2} &{}\quad - \frac{\gamma _2}{c}|t|^{\gamma _2-1} \\ - |t|^{\gamma _1} &{}\quad \frac{\gamma _1}{c}|t|^{\gamma _1-1} \end{pmatrix} \begin{pmatrix} 0 \\ {\mathcal {O}}(ct) u_1(t) \end{pmatrix}. \end{aligned}$$

Now using that ${\mathcal {O}}(ct)|u_1|\leqq Cc |t|^{1-\sigma }$, we get that

$$\begin{aligned} |\partial _t\alpha | \leqq Cc |t|^{\gamma _2-1} |t|^{1-\sigma }= Cc |t|^{\gamma _2-\sigma }, \\ |\partial _t\beta |\leqq Cc |t|^{\gamma _1-\sigma }. \end{aligned}$$

Integrating, we obtain that

$$\begin{aligned} |\alpha |\leqq C + C|t|^{1+\gamma _2-\sigma },\\ |\beta |\leqq C + C|t|^{1+\gamma _1-\sigma }. \end{aligned}$$

Plugging this into our ansatz it follows that

$$\begin{aligned} |u_1(t)|&\leqq |t|^{\gamma _1-1} (C +|t|^{1+\gamma _2-\sigma } ) + |t|^{\gamma _2-1} (C + |t|^{1+\gamma _1-\sigma }) \\&\leqq C |t|^{\gamma _2-1} + C |t|^{1-\sigma }, \\ |u_2(t)|&\leqq |t|^{\gamma _1} (C +|t|^{1+\gamma _2-\sigma } ) + |t|^{\gamma _2} (C+ |t|^{1+\gamma _1-\sigma } ) \\&\leqq C |t|^{\gamma _1} + C |t|^{2-\sigma } + C |t|^{2-\sigma }. \end{aligned}$$

Here, we used that $\gamma _1+\gamma _2=1$, but may also more roughly estimate $\gamma _1+\gamma _2\geqq 2 \gamma _2 >0$.

In particular, we observe that

$$\begin{aligned} |u_1(t)| \leqq C |t|^{\gamma _2-1} + C |t|^{2\gamma _2-\sigma } \leqq C |t|^{-\sigma } \end{aligned}$$

is a strict improvement over (48) if $\sigma > 1-\gamma _2$ initially. We may then repeat our argument with (48) with this smaller $\sigma$ and successively improve until $\sigma =1-\gamma _2$.

With this improved upper bound at hand, we now can establish the lower bounds (51). For this we observe that

$$\begin{aligned} \begin{pmatrix} \alpha (t_0) \\ \beta (t_0) \end{pmatrix}&= S(t_0)^{-1} \begin{pmatrix} u_1(t_0) \\ u_2(t_0) \end{pmatrix} \\&\approx \frac{c}{\sqrt{1-8c^2}} \begin{pmatrix} \gamma _1/c t_0^{\gamma _2-1} &{}\quad - t_0^{\gamma _2} \\ - \gamma _2/c t_0^{\gamma _1-1} &{}\quad t_0^{\gamma _1} \end{pmatrix} \begin{pmatrix} u_1(t_0) \\ u_2(t_0) \end{pmatrix}. \end{aligned}$$

Under the assumption of the lower bound on $u_2(t_0)$ it thus follows that $\beta (t_0)$ is bounded below and of comparable size to $u_2(t_0)$. It thus only remains to show that $(\alpha (t),\beta (t))$ do not deviate too much from this. Here, we use the above developed bounds on $\partial _t\alpha$ and $\partial _t\beta$ to get

$$\begin{aligned} |\partial _t\beta |&\leqq \frac{c}{\gamma } \frac{\gamma _1}{c} |t^{\gamma _1}| |u_1(t)| |t| Cc , \\ \leadsto |\beta (t)-\beta (t_0)|&= {\mathcal {O}}(c), \\ |\partial _t\alpha |&\leqq \frac{c}{\gamma } \frac{\gamma _2}{c} |t|^{\gamma _2-1} Cc |t| |u_1(t)|\\ \leadsto |\alpha (t)-\alpha (t_0)|&= {\mathcal {O}}(c^3), \end{aligned}$$

where we used that $\gamma _2=\frac{1}{2}-\sqrt{\frac{1}{4}-2 c^2}={\mathcal {O}}(c^2)$. Since *c* is small, $\alpha (t),\beta (t)$ hence indeed do not deviate too much and the result follows.

*Proof in the actual model* This proof is analogous with the only difference being that the solution matrix is now not anymore given by power laws but rather by explicit hypergeometric/Legendre functions, which are bounded above and below by such power laws.

We note that the problem

$$\begin{aligned} \partial _t\psi + \begin{pmatrix} 0 &{}\quad \frac{c}{\xi ^{-2}+t^2} \\ -2c &{}\quad 0 \end{pmatrix} \psi =0, \end{aligned}$$

can be decoupled into two second order equations and that $\psi _1$ solves

$$\begin{aligned} \partial _t(\xi ^{-2}+t^2) \partial _t\psi _1 + 2c^2 \psi _1=0. \end{aligned}$$

After some changes of coordinates one sees that this is a Legendre type equation

$$\begin{aligned} (1-t^2)y''(t) -2t y'(t)+ \nu (\nu +1)y(t)=0 \end{aligned}$$

for imaginary *t*, which has the general solution

$$\begin{aligned} \psi _1(t)= C_1 P\left( -\frac{1}{2}+\frac{1}{2}\sqrt{1-8c^2}, it \xi \right) + C_2 Q\left( -\frac{1}{2}+\frac{1}{2}\sqrt{1-8c^2}, it\xi \right) , \end{aligned}$$

where *P* is the Legendre function of the first kind and *Q* is the Legendre function of the second kind (see the NIST Digital Library of Mathematical Functions [10] https://dlmf.nist.gov/14). We here write $P(\nu , z)=P_{\nu }(z)=P_{\nu }^0(z)$). Most importantly, we are interested in the interval of *t* where $t \xi \in (c^{-1},\xi )$ is very large and thus can use the asymptotics (https://dlmf.nist.gov/14.8)

$$\begin{aligned} P(\nu , z) \sim c_{1}z^{\nu },\\ Q(\nu , z) \sim c_{2}z^{-\nu -1},\\ \end{aligned}$$

which for our purposes becomes $t^{\gamma _1-1}, t^{\gamma _2-1}$. Here, $c_{1,2}$ are explicit (if complicated) coefficients in terms of $\nu$.

The corresponding values of $\psi _2$ can then be obtained from

$$\begin{aligned} \psi _2 = - \frac{\xi ^{-2}+t^2}{c} \partial _t\psi _1, \end{aligned}$$

and the recurrence relation (see https://dlmf.nist.gov/14.10)

$$\begin{aligned} (1-x^2)\frac{\mathrm{d}}{\mathrm{d}x}P(\nu , x)= \nu P(\nu -1)(x)- \nu x P_{\nu }(x). \end{aligned}$$

Note that here $\nu \sim -\gamma _1,-\gamma _2$ was negative. Thus, for our purposes, these functions asymptotically behave as linear combinations of $(\xi t)^{\gamma _1}, (\xi t)^{\gamma _2}$.

Given these homogeneous solutions, we may thus again make the ansatz that

$$\begin{aligned} u(t)= S(t) \begin{pmatrix} \alpha (t) \\ \beta (t) \end{pmatrix}, \end{aligned}$$

where all entries of *S*(*t*) are comparable to the power laws obtained above and $\det (S(t))=\det (S(t_0))$ is bounded away from zero. We remark that the conservation of the determinant follows from

$$\begin{aligned} \partial _tS + M S =0, \end{aligned}$$

and note that the trace of *M* vanishes. $\quad \square$

Given the solution operator of problem (44), we return to problem (43) and introduce a particular solution to account for $u_3$.

##### Lemma 7

(Particular Solution) Consider the problem (43) with initial conditions

$$\begin{aligned} u_1(t_0)=u_2(t_0)=0. \end{aligned}$$

Then in the notation of Lemma 6 it holds that

$$\begin{aligned} |u_1\left( -\frac{d}{\xi }\right) |&\leqq c|u_3(t_0)| C \left( \frac{d}{\xi }\right) ^{\gamma _2-1},\\ |u_2\left( -\frac{d}{\xi }\right) |&\leqq c|u_3(t_0)| C \left( \frac{d}{\xi }\right) ^{\gamma _2}. \end{aligned}$$

An analogous result holds on $I_3=(\frac{d}{\xi },t_1)$.

##### Proof of Lemma 7

Similarly to the proof of Lemma 6 we consider a variation of constants ansatz but this time using the solution operator *S*(*t*) of the exact problem (that is, not approximating *b*). Here, we introduce

$$\begin{aligned} f= \begin{pmatrix} (b_1+b_2) u_3\\ (b_1-b_2) u_3 \end{pmatrix} \end{aligned}$$

in order to simplify notation. In particular recall that $u_3$ is conserved and hence $|f(t)|\leqq 2 c |u_3(t_0)|$ for all times.

We hence consider the ansatz

$$\begin{aligned} u(t)=S(t) \begin{pmatrix} \alpha (t) \\ \beta (t) \end{pmatrix}, \end{aligned}$$

where $\alpha (t_0)=\beta (t_0)=0$ and *S*(*t*) satisfies the power law bounds discussed in the proof of Lemma 6. In particular, $|S^{-1}(t)|\leqq C |t|^{\gamma _2-1}$ is integrable in time (by using the conserved determinant and Cramer’s rule).

Thus, it follows that

$$\begin{aligned} S(t) \partial _t\begin{pmatrix} \alpha (t) \\ \beta (t) \end{pmatrix} = f, \\ \Leftrightarrow \partial _t\begin{pmatrix} \alpha (t) \\ \beta (t) \end{pmatrix} = S(t)^{-1} f, \end{aligned}$$

and hence,

$$\begin{aligned} (\alpha (t), \beta (t)) =\int _{t_0}^t \partial _s (\alpha (s), \beta (s)) \,\mathrm{d}s \end{aligned}$$

is bounded by $$ C c |u_3(t_0)|$$. $$\quad \square $$

Finally, we return to the proof of the a priori bound by a power law (48).

##### Lemma 8

Consider the problem (44) on the interval $(t_0,-\delta )$ with $\xi ^{-1}\ll \delta \ll 1$, then there exists a constant $C>0$ and $\sigma >0$ such that

$$\begin{aligned} |u_1(t)|\leqq C |t|^{-\sigma } \Vert (u_1,u_2)(t_0)\Vert \end{aligned}$$

for all $t \in (t_0,-\delta )$. Here, *C* and $\sigma$ may depend on *c* but are uniform in $\xi$ and $\delta$.

Analogously, for the interval $(\delta ,t_1)$ it holds that

$$\begin{aligned} |u_1(t)|\leqq C |t|^{-\alpha } \Vert (u_1,u_2)(t_1)\Vert . \end{aligned}$$

##### Proof of Lemma 8

Our proof is based on concatenating estimates on a large number of very small intervals, where we can use perturbative arguments.

Thus, consider an interval $I=(t_0,t_0+\nu ) \subset (-1,\delta )$ with $t_0$ given and $\nu$ to be specified later. Then on this interval it holds that

$$\begin{aligned} |b(t)-b(t_0)| \leqq 4 c \nu . \end{aligned}$$

We thus write our system as

$$\begin{aligned} \partial _t\begin{pmatrix} u_1 \\ u_2 \end{pmatrix} + \begin{pmatrix} 0 &{}\quad a \\ - b(t_0) &{}\quad 0 \end{pmatrix} \begin{pmatrix} u_1 \\ u_2 \end{pmatrix} = \begin{pmatrix} 0 &{}\quad 0 \\ b(t)-b(t_0) &{}\quad 0 \end{pmatrix} \begin{pmatrix} u_1 \\ u_2 \end{pmatrix}. \end{aligned}$$

Considering the right-hand-side as a perturbation, we make the ansatz

52 $$\begin{aligned} \begin{pmatrix} u_1 \\ u_2 \end{pmatrix}= S(t) \begin{pmatrix} \alpha (t) \\ \beta (t) \end{pmatrix}, \end{aligned}$$

where *S*(*t*) is the solution operator to the problem with *b* replaced by $b(t_0)$. Then by Lemma 6 we know that component-wise

$$\begin{aligned} S(t)\sim \begin{pmatrix} -\frac{\gamma _1}{b(t_0)}|t|^{\gamma _1-1} &{}\quad -\frac{\gamma _2}{b(t_0)} |t|^{\gamma _2-1} \\ |t|^{\gamma _1} &{}\quad |t|^{\gamma _2} \end{pmatrix}, \end{aligned}$$

where $$\gamma _{1,2}$$ solve $$\gamma _i(\gamma _i-1)=c b(t_0)$$.

We further recall that $\det (S(t))$ is conserved due the vanishing trace in equation (44) and with the above comparison of the following size:

$$\begin{aligned} \det (S)\sim \frac{\gamma _2-\gamma _1}{b(t_0)}. \end{aligned}$$

Hence, we may easily invert this matrix using Cramer’s rule.

Plugging in this ansatz, we obtain that

$$\begin{aligned} \partial _t\begin{pmatrix} \alpha (t) \\ \beta (t) \end{pmatrix}= S(t)^{-1} \begin{pmatrix} 0 &{}\quad 0 \\ b(t)-b(t_0) &{}\quad \quad 0 \end{pmatrix} S(t) \begin{pmatrix} \alpha (t) \\ \beta (t) \end{pmatrix}. \end{aligned}$$

We now consider the associated Duhamel integral and show that for $\nu$ sufficiently small it yields a small perturbation to the identity. That is, we estimate

$$\begin{aligned}&\int _{t_0}^{t_0+\nu } \left\| S(t)^{-1} \begin{pmatrix} 0 &{}\quad 0 \\ b(t)-b(t_0) &{}\quad 0 \end{pmatrix} S(t) \right\| _{op} \,\mathrm{d}t \\&\quad \leqq \frac{b_0}{\gamma _1-\gamma _2} 4c \nu \int _{t_0}^{t_0+\nu }\left\| \begin{pmatrix} |t|^{\gamma _2} &{}\quad \frac{\gamma _2}{b(t_0)} |t|^{\gamma _2-1} \\ -|t|^{\gamma _1}&{}\quad -\frac{\gamma _1}{b(t_0)}|t|^{\gamma _1-1} \end{pmatrix} \begin{pmatrix} 0 &{}\quad 0 \\ 1 &{}\quad 0 \end{pmatrix} \begin{pmatrix} -\frac{\gamma _1}{b(t_0)}|t|^{\gamma _1-1} &{}\quad -\frac{\gamma _2}{b(t_0)} |t|^{\gamma _2-1} \\ |t|^{\gamma _1} &{}\quad |t|^{\gamma _2} \end{pmatrix} \right\| _{op}\\&\quad = \frac{b_0}{\gamma _1-\gamma _2} 4c \nu \int _{t_0}^{t_0+\nu }\left\| \begin{pmatrix} 0 &{}\quad \frac{\gamma _2}{b(t_0)} |t|^{\gamma _2-1} \\ 0 &{}\quad -\frac{\gamma _1}{b(t_0)}|t|^{\gamma _1-1} \end{pmatrix} \begin{pmatrix} 0 &{}\quad 0 \\ 1 &{}\quad 0 \end{pmatrix} \begin{pmatrix} -\frac{\gamma _1}{b(t_0)}|t|^{\gamma _1-1} &{}\quad -\frac{\gamma _2}{b(t_0)} |t|^{\gamma _2-1} \\ 0 &{}\quad 0 \end{pmatrix} \right\| _{op}. \end{aligned}$$

As $|t|<1$, we obtain an upper bound of the integrand by

$$\begin{aligned}&\frac{1}{b(t_0)^2} \left( \gamma _2^2 |t|^{2\gamma _2-2} + 2 \gamma _1 \gamma _2 |t|^{-1} + \gamma _1^2 |t|^{2\gamma _1-2}\right) \\ {}&\quad \leqq 4 c^2 |t|^{-2} + 4|t|^{-1} + 4 c^{-2} |t|^{2\gamma _2-1}, \end{aligned}$$

where we used that $c\leqq b(t_0)\leqq 4 c$, $\gamma _1+\gamma _2=1$, $\gamma _2\approx 4c^2$ (by Taylor’s approximation). Here, the last term is integrable in time, the middle term yields a logarithm and the first term is estimated rather roughly.

We may hence control

53 $$\begin{aligned}&\int _{t_0}^{t_0+\nu } \left\| S(t)^{-1} \begin{pmatrix} 0 &{}\quad 0 \\ b(t)-b(t_0) &{}\quad 0 \end{pmatrix} S(t) \right\| _{op} \end{aligned}$$

54 $$\begin{aligned}&\quad \leqq \frac{b_0}{\gamma _1-\gamma _2} 4c \nu \left( \frac{c^2}{t_0+\nu } + \ln \left( \frac{t_0+\nu }{t_0}\right) +1\right) . \end{aligned}$$

Here we used that $t_0<t_0+\nu <0$ to bound the first integral by the larger term. The case $0<t_0<t_0+\nu$ is switched accordingly. We hence note that we cannot choose $t_0+\nu$ arbitrarily small, but rather need to require that $\frac{t_0+\nu }{t_0}$ (respectively its reciprocal) is not too large. Choosing $\nu$ such that

55 $$\begin{aligned} t_0+\nu = \frac{1}{2} t_0, \end{aligned}$$

we thus obtain that (53) is bounded by $4c\ll 1$, and hence the map

$$\begin{aligned} \begin{pmatrix} \alpha (t_0) \\ \beta (t_0) \end{pmatrix} \mapsto \begin{pmatrix} \alpha (t_0+\nu ) \\ \beta (t_0+\nu ) \end{pmatrix} \end{aligned}$$

is comparable to the identity within a factor 2, provided $\nu$ satisfies (55). It thus follows that |*u*(*t*)| also only grows by a constant factor bounded by 100 on the interval $(t_0,t_0+\nu )=(t_0,t_0/2)$. In order to establish the desired bound on all of $I_1=(t_0,\delta )$, we partition our interval as

$$\begin{aligned} (t_0,t_0/2), (t_0/2,t_0/4), \ldots , (2\delta ,\delta ). \end{aligned}$$

In order to reach a given $t \in I_1$, we then concatenate $|\ln (t/t_0)|/\ln (2)$ intervals and thus obtain that

$$\begin{aligned} \Vert (u_1(t),u_2(t))\Vert \leqq 100^{|\ln (t/t_0)/\ln (2)|}\Vert (u_1,u_2)(t_0)\Vert = |t/t_0|^{-\ln (100)/\ln (2)} \Vert (u_1,u_2)(t_0)\Vert . \end{aligned}$$

This concludes the proof of our rough upper bound with $\sigma =\ln (100)/\ln (2)$. $\quad \square$

#### The Interval $I_2$

In order to study the evolution of (39) on the middle interval $I_2$ we use a different approach based on the convergent limit of iterated Duhamel integration. This method of proof also readily extends to the full (inhomogeneous) problem, which is discussed in more detail in Proposition 6. We again expect the behavior to be similar to the solution derived in Lemma 3 for the two mode model and thus exhibit growth and decay by powers $\xi ^{+1}$, $\xi ^{-1}$, as formulated in the following lemma:

##### Lemma 9

(Middle interval, homogeneous case) In the homogeneous case on the middle interval $I_2=(-\frac{d}{\xi }, \frac{d}{\xi })$, $u_3$ is again conserved and

$$\begin{aligned} \begin{pmatrix} u_1\left( \frac{d}{\xi }\right) \\ u_2\left( \frac{d}{\xi }\right) \end{pmatrix} \approx \begin{pmatrix} 1 &{}\quad C c \xi \\ \frac{2cd}{\xi } &{}\quad 1 \end{pmatrix} \begin{pmatrix} u_1\left( -\frac{d}{\xi }\right) \\ u_2\left( -\frac{d}{\xi }\right) , \end{pmatrix} \end{aligned}$$

where $$C=\arctan (\xi t)|_{-\frac{d}{\xi }}^{\frac{d}{\xi }}= 2 \arctan (d)$$.

In particular, at time $t_2=+\frac{d}{\xi }$ it holds that

$$\begin{aligned} u_1(t_2)&\approx Cc \xi u_2(t_1) \approx 2C c^{1-\gamma _2} \xi ^{1-\gamma _2} \frac{\gamma _1}{\gamma _2-\gamma _1} u_{2}(t_0), \\ u_2(t_2)&\approx u_2(t_1) \approx 2 c^{-\gamma _2} \xi ^{-\gamma _2} \frac{\gamma _1}{\gamma _2-\gamma _1} u_2(t_0). \end{aligned}$$

##### Proof

An explicit solution can be given in terms of hypergeometric functions, as we explore in the “Appendix A”. A more useful and shorter proof for our purposes considers an expansion in terms of the Duhamel iteration. Here, it turns out that the first Duhamel iteration is dominant and all higher order Duhamel iterations can be estimated in a geometric series. Since the statement of the inhomogeneous case includes this result as a special case, in order to avoid duplication, we refer to the proof of Proposition 6 for details. Concerning the asymptotics, we recall from Proposition 4 that

$$\begin{aligned} u_1(t_1)&\approx c^{2-\gamma _2} \xi ^{1-\gamma _2} u_2(t_0), \\ u_2(t_1)&\approx c^{-\gamma _2} \xi ^{-\gamma _2} u_2(t_0). \end{aligned}$$

Hence, it follows that

$$\begin{aligned}&\begin{pmatrix} 1 &{}\quad C c \xi \\ \frac{2cd}{\xi } &{}\quad 1 \end{pmatrix} \begin{pmatrix} u_1(t_1) \\ u_2(t_1), \end{pmatrix} \\&\quad \approx u_2(t_0) \begin{pmatrix} 1 &{}\quad cC \xi \\ \frac{2}{\xi } &{}\quad 1 \end{pmatrix} \begin{pmatrix} c^{2-\gamma _2} \xi ^{1-\gamma _2} \\ 2 c^{-\gamma _2} \end{pmatrix} \\&\quad \approx u_2(t_0) \begin{pmatrix} 2C c^{1-\gamma _2} \xi ^{1-\gamma _2} \\ 2 c^{-\gamma _2} \xi ^{-\gamma _2} \end{pmatrix}. \end{aligned}$$

$$\square $$

#### The Interval $I_3$

It remains to discuss the evolution on the interval $I_3=(\frac{d}{\xi },t_1)$. Since this interval roughly corresponds to a mirror image of $I_1$, we follow a similar method of proof as in Section 5.1.1, except that the time direction is in a sense reversed. For this reason, we additionally have to consider the inverse of the solution matrix.

##### Lemma 10

(Right interval, homogeneous case) Let $\xi \gg 1$ and $0<c <0.2$ be given. We consider the problem (39) on the interval $I_3=(\frac{d}{\xi },t_1)$ Then under the assumptions of Theorem 7 it holds that

$$\begin{aligned} u_1(t_1)&\approx c^{-1-\gamma _2} \xi ^{-\gamma _2}u_{1}\left( \frac{d}{\xi }\right) - c^{1-\gamma _2} \xi ^{1-\gamma _2} u_2\left( \frac{d}{\xi }\right) \approx \xi ^{\gamma } c^{1-2\gamma _2} \xi ^\gamma u_2(t_0) \\ u_2(t_1)&\approx 2c^{1-\gamma _2} \xi ^{-\gamma _2}u_1\left( \frac{d}{\xi }\right) - 2c^{2-\gamma _2} \xi ^{1-\gamma _2} u_2\left( \frac{d}{\xi }\right) \approx \xi ^{\gamma } c^{2-2\gamma _2} u_2(t_0), \\ u_3(t_1)&=u_3(t_0). \end{aligned}$$

We note that $u_1(\frac{d}{\xi })$ is multiplied by a negative power of $\xi$ while $u_2(\frac{d}{\xi })$ is multiplied with a positive power of $\xi$.

##### Proof of Lemma 10

This proof is largely analogous to the one of Proposition 4 in the sense that the inverse solution operator $u(t_1)\mapsto u(\frac{d}{\xi })$ satisfies the same (asymptotic) estimates.

As a first heuristic let us again consider the approximated two-mode model, where

$$\begin{aligned} \begin{pmatrix} u_1\left( \frac{d}{\xi }\right) \\ u_2\left( \frac{d}{\xi }\right) \end{pmatrix} \approx \begin{pmatrix} \left( \frac{d}{\xi }\right) ^{\gamma _1-1} &{}\quad \left( \frac{d}{\xi }\right) ^{\gamma _2-1}\\ \frac{\gamma _2}{c}\left( \frac{d}{\xi }\right) ^{\gamma _1} &{}\quad \frac{\gamma _1}{c}\left( \frac{d}{\xi }\right) ^{\gamma _2} \end{pmatrix} S(t_1)^{-1} \begin{pmatrix} u_1(t_1) \\ u_2(t_1) \end{pmatrix} \\ \Leftrightarrow \begin{pmatrix} u_1(t_1) \\ u_2(t_1) \end{pmatrix} = S(t_1) \begin{pmatrix} \left( \frac{d}{\xi }\right) ^{\gamma _1-1} &{}\quad \left( \frac{d}{\xi }\right) ^{\gamma _2-1}\\ \frac{\gamma _2}{c}\left( \frac{d}{\xi }\right) ^{\gamma _1} &{}\quad \frac{\gamma _1}{c}\left( \frac{d}{\xi }\right) ^{\gamma _2} \end{pmatrix}^{-1} \begin{pmatrix} u_1\left( \frac{d}{\xi }\right) \\ u_2\left( \frac{d}{\xi }\right) \end{pmatrix}. \end{aligned}$$

Now note that

$$\begin{aligned} \begin{pmatrix} \left( \frac{d}{\xi }\right) ^{\gamma _1-1} &{}\quad \left( \frac{d}{\xi }\right) ^{\gamma _2-1}\\ \frac{\gamma _2}{c}\left( \frac{d}{\xi }\right) ^{\gamma _1} &{}\quad \frac{\gamma _1}{c}\left( \frac{d}{\xi }\right) ^{\gamma _2} \end{pmatrix}^{-1} = \frac{c}{\gamma _1-\gamma _2} \begin{pmatrix} \frac{\gamma _1}{c}\left( \frac{d}{\xi }\right) ^{\gamma _2} &{}\quad - \left( \frac{d}{\xi }\right) ^{\gamma _2-1} \\ - \frac{\gamma _2}{c}\left( \frac{d}{\xi }\right) ^{\gamma _1} &{}\quad \left( \frac{d}{\xi }\right) ^{\gamma _1-1} \end{pmatrix}. \end{aligned}$$

Thus, in this model, we may compute that

$$\begin{aligned} \begin{pmatrix} u_1(t_1)\\ u_2(t_1) \end{pmatrix}&= \begin{pmatrix} t_1^{\gamma _1} &{}\quad t_1^{\gamma _2} \\ \frac{\gamma _2}{c}t_1^{\gamma _1-1} &{}\quad \frac{\gamma _1}{c} t_1^{\gamma _2-1} \end{pmatrix} \begin{pmatrix} \frac{\gamma _1}{c}\left( \frac{d}{\xi }\right) ^{\gamma _2} &{}\quad - \left( \frac{d}{\xi }\right) ^{\gamma _2-1} \\ - \frac{\gamma _2}{c}\left( \frac{d}{\xi }\right) ^{\gamma _1} &{}\quad \left( \frac{d}{\xi }\right) ^{\gamma _1-1} \end{pmatrix} \begin{pmatrix} u_1\left( \frac{d}{\xi }\right) \\ u_2\left( \frac{d}{\xi }\right) \end{pmatrix}\\&\approx \begin{pmatrix} \frac{\gamma _1}{c}\left( \frac{d}{\xi }\right) ^{\gamma _2} &{}\quad -\left( \frac{d}{\xi }\right) ^{\gamma _2-1} \\ 2c \left( \frac{d}{\xi }\right) ^{\gamma _2} &{}\quad -\frac{\gamma _2}{c} \left( \frac{d}{\xi }\right) ^{\gamma _2-1} \end{pmatrix} \begin{pmatrix} u_1\left( \frac{d}{\xi }\right) \\ u_2\left( \frac{d}{\xi }\right) \end{pmatrix}\\&=\begin{pmatrix} \frac{\gamma _1}{c}d^{\gamma _2} &{}\quad -d^{\gamma _2-1} \\ 2c \ d^{\gamma _2} &{}\quad -\frac{\gamma _2}{c} d^{\gamma _2-1} \end{pmatrix} \begin{pmatrix} \xi ^{-\gamma _2} u_1\left( \frac{d}{\xi }\right) \\ \xi ^{1-\gamma _2}u_2\left( \frac{d}{\xi }\right) \end{pmatrix}\\&\approx \begin{pmatrix} \frac{\gamma _1}{c}d^{\gamma _2} &{}\quad -d^{\gamma _2-1} \\ 2c \ d^{\gamma _2} &{}\quad -\frac{\gamma _2}{c} d^{\gamma _2-1} \end{pmatrix} \begin{pmatrix} 2C c^{1-\gamma _2} \frac{\gamma _1}{\gamma _2-\gamma _1} \\ 2 c^{-\gamma _2} \frac{\gamma _1}{\gamma _2-\gamma _1} \end{pmatrix} \xi ^{\gamma } u_2(t_0), \end{aligned}$$

where we considered smaller powers of $\xi$ as errors and denoted $\gamma =1-2\gamma _2=2\sqrt{\frac{1}{4}-2c^2}=\sqrt{1-8c^2}$. Choosing $d=c^{-1}$ and recalling that $\gamma _2\approx 2c^2$ by Taylor’s approximation, $\gamma _1 \approx \gamma \approx 1$, and we may further approximate to get

$$\begin{aligned}&\begin{pmatrix} \frac{\gamma _1}{c}d^{\gamma _2} &{}\quad -d^{\gamma _2-1} \\ 2c \ d^{\gamma _2} &{}\quad -\frac{\gamma _2}{c} d^{\gamma _2-1} \end{pmatrix} \begin{pmatrix} 2C c^{1-\gamma _2} \frac{\gamma _1}{\gamma _2-\gamma _1} \\ 2c^{-\gamma _2} \frac{\gamma _1}{\gamma _2-\gamma _1} \end{pmatrix} \\&\quad \approx \begin{pmatrix} c^{-1-\gamma _2} &{}\quad -c^{1-\gamma _2} \\ 2 c^{1-\gamma _2} &{}\quad - 2c^{2-\gamma _2} \end{pmatrix} \begin{pmatrix} 2C c^{1-\gamma _2} \\ 2c^{-\gamma _2} \end{pmatrix}\\&\quad = \begin{pmatrix} C c^{2\gamma _2} - 2 c^{1-2\gamma _2} \\ 2C c^{2-2\gamma _2} - 4 c^{2-2 \gamma _2} \end{pmatrix} \approx \begin{pmatrix} -2 c^{1-2 \gamma _2} \\ {\tilde{C}} c^{2-2 \gamma _2} \end{pmatrix}. \end{aligned}$$

In particular, we stress that $u_1(t_1)\geqq c^{-1} u_{2}(t_1)$ is much bigger provided that *c* is sufficiently small.

It remains to discuss the extension to the full problem. We note that under the transformation $(u_1(t),u_2(t),u_3(t))\mapsto (-u_1(-t),u_2(-t),u_3(-))$ we obtain an analogous problem as the one considered on $I_1$ in Section 5.1.1 except that $t_0$ is replaced by $-t_1$ and *b*(*t*) is replaced by $b(-t)$. By the same arguments as in Section 5.1.1 it hence follows that the solution operator $u(t):=S(t)u(t_1)$ is of the form

$$\begin{aligned} S= \begin{pmatrix} S' &{} \begin{matrix} b \\ c \end{matrix}\\ \begin{matrix} 0 &{}\quad 0 \end{matrix}&1 \end{pmatrix}, \end{aligned}$$

where $S'$ is asymptotically well-approximated by our two-mode model heuristic.

We may then explicitly compute the inverse of *S* as

$$\begin{aligned} S(t)^{-1}= \begin{pmatrix} S'^{-1} &{} \begin{matrix} {\tilde{b}} \\ {\tilde{c}} \end{matrix}\\ \begin{matrix} 0 &{}\quad 0 \end{matrix}&1 \end{pmatrix}, \\ \begin{pmatrix} {\tilde{b}} \\ {\tilde{c}} \end{pmatrix} = - S'^{-1} \begin{pmatrix} b \\ c \end{pmatrix}, \end{aligned}$$

where we can use Cramer’s rule for inverting *S*. As we know that $S(\frac{d}{\xi })$ is well-approximated by the power laws the result for the full model hence follows by the exact same argument as we discussed for the approximate model above. $\quad \square$

In the following section we use multiple bootstrap arguments to show that this behavior persists in the full problem.

### The Full Model

Building on the results for the three-mode model of Section 5.1 we show in the following that the full dynamics have the same asymptotic behavior. In particular, this shows that if the logarithmic constraint imposed in prior works is removed this imposes not only technical challenges but results in different asymptotics. In Section 5.3 we crucially rely on this improvement to construct solutions with initial data in Gevrey regularity which exhibit infinitely many cascades of arbitrarily long length. Furthermore, they *diverge* in (any) Sobolev regularity but their force field nevertheless *converges*. Damping persists despite the failure of scattering. The estimates for a single resonance in the full problem are summarized in the following theorem:

#### Theorem 8

(Summary) Let $c \in {\mathbb {R}}$ with $0<|c|<0.1$ and let $\xi \gg 1$. Let *u* be the solution of the full model (34):

$$\begin{aligned} \partial _tu(k)+ a(k+1) u(k+1) - a(k-1)u(k-1)=0, \end{aligned}$$

on $(t_0,t_1)$. Suppose that at time $t_0$ it holds that $u(k_0)\geqq 0.5 \max |u|=:0.5 \theta$ and define $\gamma =\sqrt{1-8c^2}$. Then there exists a constant $C>0$ such that it holds that, at time $t=t_1$,

$$\begin{aligned} |u(k)| \leqq C \xi ^{\gamma } \theta \end{aligned}$$

for all *k* and

$$\begin{aligned} |u(k_0- 1)| \geqq \frac{C}{2} \xi ^{\gamma } \theta . \end{aligned}$$

In particular, *u* at time $t_1$ satisfies the assumptions imposed at time $t_0$. We may therefore apply this theorem again on the next time interval.

As in Section 5.1 we split our proof into considering the three intervals $I_1,I_2,I_3$ and then combining the respective estimates to establish Theorem 8. On the intervals $I_1$ and $I_3$ we employ a bootstrap approach and a variation of constants ansatz to control the effect of non-resonant modes. In the center interval $I_2$ we instead are able to explicitly construct a solution operator by Duhamel iteration. We stress that if the logarithmic smallness condition is imposed this approach can be used on the full interval (see Section 3) but that this cannot work without this constraint. Indeed, higher order Duhamel iterations are much larger (by powers of logarithms) than the first iteration and their cancellations yield the modified exponent.

#### The Interval $I_1$

As in Section 5.1.1, our main aim of this section is to show that on the interval $I_1$ now for the full solution the mode $u(k_0\pm 1)$ increases with a power law $\xi ^{1-\gamma _2}$, while $u(k_0)$ decreases with a power law $\xi ^{-\gamma _2}$, with $\gamma _2$ as in Theorem 8. Compared to Proposition 4 a main challenge here is given by the coupling to other modes than $k_0-1,k_0,k_0+1$, which appear as a right-hand-side in the inhomogeneous problem and themselves evolve. Our strategy in the following is thus to assume that we can control all modes in an appropriate way at least for some small positive time, which is quantified in the bootstrap ansatz (59) to (63). Using the equations we then show that all controls improve on that time interval and hence must remain valid on the whole interval $I_1$. We summarize our results in the following proposition:

##### Proposition 5

(Left interval, inhomogeneous case) Let $c \in {\mathbb {R}}$ with $0<|c|<0.1$ and let $\xi \gg 1$. Let *u* be as in Theorem 8. Then at $-\frac{d}{\xi }=-\frac{1}{c\xi }$ it holds that

56 $$\begin{aligned} u(k_0)&\approx \theta \ c \ \left( \frac{d}{\xi }\right) ^{\gamma _2}, \end{aligned}$$

57 $$\begin{aligned} u(k_0\pm 1)&\approx \theta \ c \ \left( \frac{d}{\xi }\right) ^{\gamma _2-1}, \end{aligned}$$

58 $$\begin{aligned} u(k)&\leqq C \theta , \end{aligned}$$

where $$C=(e^{c}-1)$$

##### Proof

By linearity without loss of generality we may set $\theta =1$.

Then at least for small positive time it holds that

59 $$\begin{aligned} u(k_0)&\approx c u_2(t_0) |t|^{\gamma _2}, \end{aligned}$$

60 $$\begin{aligned} (u(k_0+1)- u(k_0-1))&\approx c u_2(t_0) |t|^{\gamma _2-1}, \end{aligned}$$

61 $$\begin{aligned} \left( \frac{u(k_0+1)+ u(k_0-1)}{2}\right) _{t_0}^t&\leqq e^{4.1 c(t-t_0)} -1 + (t^{\gamma _{2}}-t_0^{\gamma _{2}}), \end{aligned}$$

62 $$\begin{aligned} u(k)&\leqq e^{2.1 c(t+1)} -1 + C (t^{\gamma _{2}}-t_0^{\gamma _{2}})\text { else}, \end{aligned}$$

63 $$\begin{aligned} \Vert u \ 1_{|k-k_0|\geqq 2}\Vert _{l^2}(t)&\leqq e^{4.1 c(t-t_0)} \Vert u \ 1_{|k-k_0|\geqq 2}\Vert _{l^2}(t_0). \end{aligned}$$

Here $1_{|k-k_0|\geqq 2}(k) \in \{0,1\}$ denotes the characteristic function and we denoted $u_2(t_0)=u(k_0)|_{t=t_0}$ in analogy to the notation of the homogeneous model of Section 5.1.

In what follows we argue by bootstrap that the maximal time satisfying these estimates is given by $T=\frac{1}{c\xi }$.

Indeed, suppose there were $T<\frac{1}{c\xi }$ maximal with these properties. We then show that all conditions (59) to (62) do not achieve equality at time *T* and that hence *T* could be chosen larger by continuity, which contradicts the maximality.

*Ad* (61) In a fashion similar as to Lemma 5, we may use the symmetry of the problem to compute that

$$\begin{aligned} \partial _t(u(k_0+1)+ u(k_0-1))&= -a(k_0+2)u(k_0+2)+ a(k_0-2) u(k_0-2))\\&\Rightarrow |u(k_0+1)+ u(k_0-1)||_{t_0}^{t} \leqq c \int _{t_0}^{T} (|u(k_0+2)|\\&\quad + |u(k_0-2)|) \\&\leqq 2c \int _{t_0}^{T} e^{4.1 c(t-t_0)} +\left( t^{\gamma _{2}}-t_0^{\gamma _{2}}\right) . \end{aligned}$$

We may then roughly control $|t^{\gamma _{2}}-t_0^{\gamma _{2}}|\leqq 1 \leqq e^{4.1 c(t-t_0)}$ and thus control the integral by

$$\begin{aligned} \frac{4}{4.1} \left( e^{2.1c(T-t_0)}-1\right) <\left( e^{4.1c(T-t_0)}-1\right) . \end{aligned}$$

Thus, equality in (61) is not attained at time *T*.

*Ad* (62) First suppose in addition that $k\ne k_0+2, k_0-2$. Then it holds that

$$\begin{aligned} u(k)|_{t_0}^T&= \int _{t_0}^T -a(k+1) u(k+1) + a(k-1) u(k-1) \leqq 4c \int _{t_0}^T e^{4.1 c(t-t_0)} \,\mathrm{d}t\\&= \frac{4}{4.1} \left( e^{2c(T-t_0)}-1\right) <e^{2c(T-t_0)}-1, \end{aligned}$$

where we again estimated $|t^{\gamma _{2}}-t_0^{\gamma _{2}}|\leqq 1 \leqq e^{4.1 c(t-t_0)}$ and used that $|a(k)|\leqq c$ unless $k=k_0$.

If $k=k_0\pm 2$, $\int _{t_0}^T \partial _t u(k)$ additionally involves

$$\begin{aligned} \int _{t_0}^T a(k_0\pm 1) u(k_0\pm 1) \,\mathrm{d}t \leqq c t^{\gamma _{1,2}}|_{t_0}^T, \end{aligned}$$

which is controlled by $t^{\gamma _{1,2}}-t_0^{\gamma _{1,2}}$.

*Ad* (63) Taking a time-derivative of the energy, that is the left-hand-side, we get that

$$\begin{aligned} \partial _tE(t)\leqq 4c E(t) + c|u(k_0+2)||u(k_0+1)| \leqq 4c E(t) + c (e^{4.1ct}+2)(t^{\gamma _{2}}-t_0^{\gamma _{2}}). \end{aligned}$$

The estimate hence follows by Gronwall’s lemma or by multiplying with $e^{-4ct}$ and then integrating.

The main part of the proof is thus given by the proof of the estimates for $u(k_0)$ and $u(k_0+1)-u(k_0-1)$.

*Ad* (59) *and* (60) Following a similar argument as in the proof of Proposition 4, we study the inhomogeneous problem for $(\frac{u(k_0+1)-u(k_0-1)}{2}, u(k_0))=: (u_1,u_2)$:

$$\begin{aligned} \partial _t\begin{pmatrix} u_1 \\ u_2 \end{pmatrix} + \begin{pmatrix} 0 &{}\quad a \\ b &{}\quad 0 \end{pmatrix} \begin{pmatrix} u_1 \\ u_2 \end{pmatrix}&=- \begin{pmatrix} a(k_0+2)u(k_0+2)/2- a(k_0-2)u(k_0-2)/2 \\ (a(k_0+1)-a(k_0-1)) (u(k_0+1)+u(k_0-1))/2 \end{pmatrix}\\&=: f. \end{aligned}$$

As in Lemma 6 we make a variation of constants ansatz, where for simplicity of notation write our calculations in terms of the power law solutions of the approximate model. By the estimates of Section 5.1, the solution operator *S*(*t*) of the full model is comparable, and hence the exact same proof immediately applies:

$$\begin{aligned} \begin{pmatrix} u_1 \\ u_2 \end{pmatrix} = S(t) \begin{pmatrix} \alpha (t) \\ \beta (t) \end{pmatrix} \approx \alpha (t) \begin{pmatrix} t^{\gamma _1-1}\\ t^{\gamma _1}\frac{\gamma _2}{c} \end{pmatrix} + \beta (t) \begin{pmatrix} t^{\gamma _2-1}\\ t^{\gamma _2}\frac{\gamma _1}{c} \end{pmatrix}. \end{aligned}$$

Solving at time $t_0$, we obtain

$$\begin{aligned} \begin{pmatrix} \alpha (t_0)\\ \beta (t_0) \end{pmatrix}&= S(t_0)^{-1} \begin{pmatrix} u_1(t_0) \\ u_2(t_0) \end{pmatrix} \\&\approx \frac{1}{\sqrt{1-8c^2}} \begin{pmatrix} \gamma _1t_0^{\gamma _2-1} &{}\quad -c t_0^{\gamma _2} \\ - \gamma _2 t_0^{\gamma _1-1} &{}\quad ct_0^{\gamma _1} \end{pmatrix} \begin{pmatrix} u_1(t_0) \\ u_2(t_0) \end{pmatrix}. \end{aligned}$$

Here, in the proof of Proposition 4 we concluded by noting that only $\beta (t_1)=\beta (t_1)\approx c u_2(t_0)$ is relevant at $t_1$ (due to the smaller powers $\xi$ for $\alpha$). In order to establish the desired bounds in this inhomogeneous case, we hence need to show that $\alpha , \beta$ do not deviate from this too much. We compute

$$\begin{aligned} \partial _t\begin{pmatrix} \alpha \\ \beta \end{pmatrix}&= -c \begin{pmatrix} t^{\gamma _1-1} &{}\quad t^{\gamma _2-1}\\ t^{\gamma _1}\frac{\gamma _2}{c} &{}\quad t^{\gamma _2}\frac{\gamma _1}{c} \end{pmatrix}^{-1} f\\&= -\frac{c^2}{\sqrt{1-8c^2}} \begin{pmatrix} t^{\gamma _2}\frac{\gamma _1}{c} &{}\quad - t^{\gamma _2-1}\\ - t^{\gamma _1}\frac{\gamma _2}{c} &{}\quad t^{\gamma _1-1} \end{pmatrix} f. \end{aligned}$$

By estimates (62) and (61) we note that $|f|\leqq c$ and we further note that the integrals of $t^{\gamma _1}, t^{\gamma _1-1}$ are uniformly bounded, while $\int t^{\gamma _2-1}\leqq \frac{1}{\gamma _2}\leqq c^{-2}$, where we used that $\gamma _2 \approx 2 c^2$. Hence, in total it follows that $(\alpha , \beta )|_{t_0}^t \leqq C (c,c^3)$ for all times and therefore $(\alpha (t),\beta (t))\approx (\alpha (t_0), \beta (t_0))$, which implies the result. $\quad \square$

#### The Interval $I_2$

As in Section 5.1.2, we next study the evolution, now of the full solution, on the middle interval $I_2=(-\frac{d}{\xi }, \frac{d}{\xi })$. Here again our main aim is to quantify the growth and decay of the three modes $k_0+1,k_0,k_0-1$ and control all other modes. In particular, we again are focused on the power law dependence in terms $\xi$ for $\xi$ large, which arises from the evolution on $I_2$ and the one on $I_1$ studied in the previous section.

Since the interval $I_2$ is shorter the larger $\xi$ is, in what follows we rely on a characterization of the solution in terms of a Duhamel series (see also Section 3 for the associated notion of paths). We then first study the evolution for initial data concentrated on the resonant mode $k_0$ in Lemma 11, which includes with a power law $\xi ^1$. If the initial data is instead concentrated on a non-resonant mode as in Lemma 12 the contribution is small. Combining both cases and using the linearity of the solution operator in the initial data, we obtain the following main proposition of this subsection:

##### Proposition 6

(Middle interval, inhomogeneous case) Let *u* be as in Theorem 8. Then at time $\frac{d}{\xi }$ it holds that

$$\begin{aligned} u(k_0\pm 1)|_{-d/\xi }^{d/\xi }&\approx C c\xi u(k_0,t_1) \approx \theta \ C c^{1-\gamma _2} \xi ^{1-\gamma _2} , \\ u(k_0)|_{-d/\xi }^{d/\xi }&\approx \frac{c}{\xi } u(k_0\pm 1, t_1) \approx \theta \ c^{-\gamma _2} \xi ^{-\gamma _2},\\ u(k)|_{-d/\xi }^{d/\xi }&\leqq \frac{cd}{\xi } u(k_0\pm 1, t_1) \leqq \theta \ c^{1-\gamma _2} \xi ^{1-\gamma _2} , \end{aligned}$$

where $$C=2 \arctan (d)\approx \pi $$.

We here use the Duhamel iteration, which we phrase as properties of the solution map for single mode initial data. Arbitrary initial data can then be realized as a linear combination. We emphasize that on this short time interval the action $k_0 \mapsto k_0\pm 1$ is large (of size $C c \xi$), while all other actions are small perturbations of the identity.

##### Lemma 11

Suppose that at time $-\frac{d}{\xi }$ it holds that $u(k)=\delta _{k k_0}$. Then at time $\frac{d}{\xi }$, *u* satisfies

1. $$|u(k_0)-1|\leqq \frac{1}{1-c_1}\frac{c_2}{1-c_2}$$,
2. $$|u(k_0\pm 1)\mp 2c \xi \arctan (\xi t)|_{-d/\xi }^{d/\xi }|\leqq \frac{2c}{1-4c^2d} 2c \xi \arctan (\xi t)|_{t_0}^{t_1}$$,
3. $$|u(k)|\leqq |\frac{c}{\xi }|^{|k-k_0|-1}\frac{1}{1-c}$$ else,

where $$c_1=\frac{4cd}{\xi }=\frac{4}{\xi }$$ and $$c_2=8 c^2 d\arctan (\xi t)_{t_1}^{t_2}= 8c \arctan (d)\approx 4 \pi c$$.

##### Proof

*Ad* (i) Let $\gamma =(k_0,\ldots , k_0)$ be a path starting and ending in $k_0$, then we may roughly estimate

$$\begin{aligned} \iint _{t_0\leqq \tau _1\leqq \cdots \leqq t_1} \prod _{i} a(\gamma _i,\tau _i) \text {sgn}(\gamma _{i+1}-\gamma _{i}) \,\mathrm{d}\tau _i \end{aligned}$$

by

$$\begin{aligned} \prod \Vert a(\gamma _i,\tau _i)\Vert _{L^1([-d/\xi ,d/\xi ])} = \left( \frac{2cd}{\xi }\right) ^{j_1} \left( c \xi \arctan (\xi t)|_{-d/\xi }^{d/\xi }\right) ^{j_2}, \end{aligned}$$

where $j_1$ corresponds to the number of non-resonance and $j_2$ to the number of resonances $\gamma _i=k_0$ (recall that the last entry of $\gamma$ does not appear in the integral). Then in order to start end in $k_0$ it needs to hold that $j_1\geqq j_2$, since we have to come back to $k_0$ before leaving it again. Thus, the contribution of the path $\gamma$ is controlled by

$$\begin{aligned} \left( \frac{2cd}{\xi }\right) ^{j_1-j_2} \left( 2 c^2 d \arctan (\xi t)|_{-d/\xi }^{d/\xi }\right) ^{j_2}. \end{aligned}$$

Estimating the number of paths of given length $j_1+j_2$ by $2^{j_1+j_2}$ the sum over the integrals of all such paths can be controlled by

$$\begin{aligned} \frac{1}{1-\frac{4cd}{\xi }} \frac{8c^2d \arctan (\xi t)|_{-d/\xi }^{d/\xi }}{1-8c^2d \arctan (\xi t)|_{-d/\xi }^{d/\xi }}, \end{aligned}$$

where we used that $j_2\geqq 1$.

*Ad* (ii) Let us first consider the special paths $\gamma =(k_0,k_0\pm 1)$, which yield an integral

$$\begin{aligned} \mp \int _{-d/\xi }^{d/\xi } a(k_0) \,\mathrm{d}\tau = \mp c \xi \arctan (\xi t)|_{-d/\xi }^{d/\xi }\gg 1. \end{aligned}$$

For any other paths $\gamma$ starting in $k_0$ and ending in $k_0\pm 1$, we may again roughly bound the integral by

$$\begin{aligned} \left( \frac{2cd}{\xi }\right) ^{j_1} \left( c \xi \arctan (\xi t)|_{-d/\xi }^{d/\xi }\right) ^{j_2}, \end{aligned}$$

where now $j_1\geqq j_2-1\geqq 0$ and we already treated $j_2=1,j_1=0$ separately. We may thus express this bound as

$$\begin{aligned} \left( \frac{2cd}{\xi }\right) ^{{\hat{j}}_1} \left( 2 c^2 d \arctan (\xi t)|_{-d/\xi }^{d/\xi }\right) ^{{\hat{j}}_2} \left( c \xi \arctan (\xi t)|_{-d/\xi }^{d/\xi }\right) , \end{aligned}$$

where $({\hat{j}}_1,{\hat{j}}_2)=(0,0)$ is excluded. Again estimating the number of such paths from above by $2^{{\hat{j}}_1+{\hat{j}}_2+1}$ and summing the geometric series, we obtain the desired result.

*Ad* (iii) Let $k \not \in \{k_0-1,k_0,k_0+1\}$. Then given a path $\gamma =(k_0, \ldots , k)$ there is a last time where $\gamma _i=k_0$, after which the remainder path is non-resonant at least $|k-k_0|$ times. Grouping all paths with the same remainder, we first estimate the contribution by the segments up to the last resonance as in (i) by

$$\begin{aligned} \frac{1}{1-\frac{4cd}{\xi }} \frac{8c^2d \arctan (\xi t)|_{-d/\xi }^{d/\xi }}{1-8c^2d \arctan (\xi t)|_{-d/\xi }^{d/\xi }} \end{aligned}$$

and then estimate the sum over all possible remainders by

$$\begin{aligned} \sum _{j\geqq |k-k_0|} \left( \frac{4cd}{\xi }\right) ^{j}= \frac{1}{1-\frac{4cd}{\xi }} \left( \frac{4cd}{\xi }\right) ^{|k-k_0|}, \end{aligned}$$

where we again introduced a factor $2^j$ to account for the number of all remainders of a given length. $\quad \square$

##### Lemma 12

Let $l \ne k_0$ and suppose that at time $-\frac{d}{\xi }$ it holds that $u(k)=\delta _{k l}$. Then *u* satisfies

$$\begin{aligned} u(k)|_{-d/\xi }^{d/\xi }&\leqq \left( \frac{4cd}{\xi }\right) ^{|k-l|} + \left( \frac{4cd}{\xi }\right) ^{|k-k_0|+|l-k_0|-1} \left( 8c^2 d\arctan (t \xi )|_{-d/\xi }^{d/\xi }\right) \\&\quad \times \frac{1}{(1-\frac{4cd}{\xi })^2} \frac{1}{1-8c^2d\arctan (t\xi )|_{-d/\xi }^{d/\xi }}. \end{aligned}$$

Here, with slight abuse of notation $|a-b|$ denotes the minimal path length between *a* and *b*, i.e. $|a-a|=2$.

##### Proof

Let $k\in {\mathbb {Z}}$ be given. Consider first the case of a purely non-resonant path $\gamma$, which can be estimated by

$$\begin{aligned} \left( \frac{2cd}{\xi }\right) ^{|\gamma |}. \end{aligned}$$

Any such path has length at least $|k-l|$ (with $|l-l|=2$). The first summand in the above estimate is hence obtained by a geometric series.

Next consider a path with at least one resonance. We group all paths that share the segment from first to last resonance and observe that at least $|l-k_0|$ non-resonances are needed to reach $k_0$ for the first time and at least $|k-k_0|-1$ non-resonances to reach *k* from $k_0$. We then may again control the sum over all path segments from $k_0$ to $k_0$ as in the previous lemma and control the first and last segment by a geometric series. $\quad \square$

Using the result of the preceding lemmas, we can now prove Proposition 6.

##### Proof of Proposition 6

By linearity we may decompose our initial data $u(t_1)$ as

$$\begin{aligned} u(t_1)= u(t_1)|_{k=k_0} + u(t_1)|_{k\in \{k_0-1,k_0+1\}} + u^r(t_1). \end{aligned}$$

Then by Lemma 12 and the bound on $u^r(t_1)$ established in Proposition 5, we may estimate its contribution to $u(t_2)$ by

$$\begin{aligned} \left( \left( \frac{4}{\xi }\right) ^{|\cdot |} * u^r(t_1)\right) (l) + Cc \left( \frac{4}{\xi }\right) ^{|l-k_0|}\left( \left( \frac{4}{\xi }\right) ^{|\cdot -k_0|-1} * u^r(t_1)\right) (l), \end{aligned}$$

where $C=\frac{16\arctan (d)}{(1-\frac{4}{\xi })^2(1-16c\arctan (d))}$. This contribution is thus very smooth and small and can be considered a perturbation.

Concerning the contributions by $k_0$ and $k_0\pm 1$, we note that Lemmas 11 and 12 combined with the bounds on $u(t_1)$ established in Proposition 5 control *u*(*k*) for $k \in \{k_0-1,k_0,k_0+1\}$ in the desired way and show that

$$\begin{aligned} \begin{pmatrix} \frac{u(k_0+1)-u(k_0-1)}{2}\\ u(k_0) \end{pmatrix}|_{t=t_2} \approx \begin{pmatrix} 1 &{}\quad 2Cc \xi \\ \frac{1}{\xi } &{}\quad 1 \end{pmatrix} \begin{pmatrix} \frac{u(k_0+1)-u(k_0-1)}{2}\\ u(k_0) \end{pmatrix}|_{t=t_1}. \end{aligned}$$

We thus conclude as in the proof of Lemma 9. $\quad \square$

#### The Interval $I_3$

It remains to control the evolution of the full equation on the right interval $I_3$, where we are gain focused on the power law dependence in terms of $\xi$. Combining the growth and decay behavior on this interval with the descriptions derived in the previous subsections, we can then conclude our proof of Theorem 8 and thus obtain a description of the growth of our solution on the full resonant interval $I_1 \cup I_2 \cup I_3$. Subsequently, in Section 5.3, we combine several of these intervals (for different *k*) to construct chains of resonance and associated solutions exhibiting norm inflation. Furthermore, we combine countably many chains to construct solutions with non-trivial asymptotic behavior.

Similarly to Section 5.1.3 we observe that the right interval is basically a mirror image of the left interval $I_1$ and can hence be treated in a similar way as in Section 5.2.1 except that the time direction is in a sense reversed. Thus, we additionally have to control inverses of the solution operator.

##### Proposition 7

(Right interval, inhomogeneous case) Let *u* be as in Theorem 8. Then it holds that at time $t_1$, all modes satisfy

$$\begin{aligned} u\leqq C \xi ^{\gamma }, \end{aligned}$$

and $u(k_0\pm 1)$ is bounded below by $\frac{C}{2}\xi ^{\gamma }$.

##### Proof

We again make a bootstrap ansatz similar to the one in Proposition  5, but now take into account the different powers of $\xi$ on different modes:

64 $$\begin{aligned} u(k_0+1)- u(k_0-1)&\approx 2 \theta c \xi ^{\gamma } t^{1-\gamma _2} \end{aligned}$$

65 $$\begin{aligned} u(k_0)&\approx \theta |\xi |^{\gamma } t^{\gamma _1}, \end{aligned}$$

66 $$\begin{aligned} u(k_0+1)+ u(k_0-1)|_{d/\xi }^t&\leqq \xi ^{\gamma } (e^{4.1c(t-d/\xi )}-1 + t^{\gamma _{1}}-d/\xi ^{\gamma _1}), \end{aligned}$$

67 $$\begin{aligned} u(k)&\leqq \xi ^{\gamma } (e^{4.1c(t-d/\xi )}-1+t^{\gamma _{1}}-d/\xi ^{\gamma _1}) \text { else}. \end{aligned}$$

Ad (67) and (66): Here, we again use that $u(k),\frac{u(k_0+1)+u(k_0-1)}{2} \leqq \xi ^{\gamma }$ at time $d/\xi$ and thus estimate

$$\begin{aligned} \int _{d/\xi }^t \partial _tu \leqq 2c \xi ^{\gamma } e^{4.1c(t+1)}<\xi ^{\gamma } (e^{4.1c(t+1)}-1), \end{aligned}$$

where we roughly estimate $t^{\gamma _{1}}-d/\xi ^{\gamma _1}\leqq 1 \leqq e^{4.1c(t-d/\xi )}$ again.

Ad (64) and (65): We make the same variation of constants ansatz as in the proof of Proposition 5 and again for simplicity of notation write the power law approximate solutions instead. Thus consider

$$\begin{aligned} \begin{pmatrix} u_1 \\ u_2 \end{pmatrix} = S(t) \begin{pmatrix} \alpha (t) \\ \beta (t) \end{pmatrix} \approx \alpha (t) \begin{pmatrix} t^{\gamma _1-1}\\ t^{\gamma _1}\frac{\gamma _2}{c} \end{pmatrix} + \beta (t) \begin{pmatrix} t^{\gamma _2-1}\\ t^{\gamma _2}\frac{\gamma _1}{c} \end{pmatrix}. \end{aligned}$$

Here, as in the proof of 6 in the following we present our argument in terms the approximate coefficients for simplicity of notation.

We first compute $\alpha ,\beta |_{d/\xi }$ by solving

$$\begin{aligned} \begin{pmatrix} \alpha \\ \beta \end{pmatrix}|_{t=\frac{d}{\xi }} = \begin{pmatrix} t^{\gamma _2}\frac{\gamma _1}{c} &{}\quad - t^{\gamma _2-1}\\ -t^{\gamma _1}\frac{\gamma _2}{c} &{}\quad t^{\gamma _1-1} \end{pmatrix}|_{t=\frac{d}{\xi }} \begin{pmatrix} u_1 \\ u_2 \end{pmatrix}|_{t=\frac{d}{\xi }}. \end{aligned}$$

Plugging in the relations between $\xi ^{-\gamma _2} u_1(t_2)$ and $\xi ^{1-\gamma _2} u_2(t_2)$, we see that this contribution satisfies the claimed estimates.

It hence remains again to study the perturbations due to the inhomogeneity. As in the proof of Proposition 5 we compute

$$\begin{aligned} \partial _t \begin{pmatrix} \alpha \\ \beta \end{pmatrix} = -c \begin{pmatrix} t^{\gamma _1-1} &{}\quad t^{\gamma _2-1} \\ -t^{\gamma _1}\frac{\gamma _2}{c} &{}\quad t^{\gamma _2}\frac{\gamma _1}{c} \end{pmatrix}^{-1} f. \end{aligned}$$

Plugging in our bounds (66) and (67) by $C \xi ^{\gamma }$ into *f* and integrating it follows that

$$\begin{aligned} \left| \begin{pmatrix} \alpha \\ \beta \end{pmatrix}|_{t=\frac{d}{\xi }}^{t} \right| \ll c \xi ^{\gamma }. \end{aligned}$$

Thus, the value of $(\alpha ,\beta )$ at time $\frac{d}{\xi }$ is dominant and satisfies the estimates. $\quad \square$

This concludes the proof of Theorem 8, which established growth of certain modes of our solution around the time $\frac{\eta }{k}$ with a rate proportional to $(\frac{\xi }{k^2})^{\gamma }$. As sketched in Section 3 in the following section we connect this growth on subsequent time intervals (for $k, k-1,k-2,\ldots$) to form an echo chain, which exhibits norm inflation. Furthermore, since we do not require any smallness condition on $\eta$ unlike prior works we can can consider $\xi$ arbitrarily large. Furthermore, using the linearity of the equation and considering families of frequency which are well separated, we can construct solutions which exhibit infinitely many echo chains of increasing lengths. In particular, these solutions do not become trivial after a final resonant time and can hence exhibit non-trivial asymptotic behavior like blow-up.

### Modified Scattering and Inviscid Damping

In the preceding sections we have shown that the linearized Euler equations (7) exhibit resonances due to the Orr mechanism. In particular, we showed that the logarithmic smallness constraint imposed in prior works is not only a technical restriction but that for large frequencies the asymptotics differ greatly, which manifests in a modified exponent. Furthermore, removing this constraint allows us to consider initial data which is *not* compactly supported in Fourier space.

As our main result we thus show that the linearized Euler equations not only exhibit full echo chains and the associated Gevrey norm inflation but that for data in a critical Gevrey regularity class we obtain blow-up and damping at the same time. That is, we construct smooth initial data $\omega _0\in {\mathcal {G}}_{C, \frac{1}{2}}$ which exhibits infinitely many resonance chains of arbitrarily long length. For this solution any Sobolev norm $\Vert \omega (t)\Vert _{H^s}, s\geqq 0$ *diverges* to infinity as time tends to infinity but despite this the associated velocity perturbation *converges*. Linear inviscid damping persists despite the failure of scattering and nearby traveling wave-like solutions are the underlying cause of Gevrey blow-up! We stress that one of the main challenges we had to overcome in this work is that one has to be able to consider infinitely many echo chains of increasing length. In particular, one has to allow for arbitrarily large frequencies, while prior works had imposed a logarithmic smallness constraint.

#### Theorem 9

(Modified Scattering) Let $0<c<0.2$ be given. Then there exists $C_0=C_0(c)$ such that if $\omega _0 \in {\mathcal {G}}_{C, \frac{1}{2}}$ with $C>C_0$ then $\omega (t) \in {\mathcal {G}}_{C-C_0, \frac{1}{2}}$ globally in time and *u*(*t*) converges in ${\mathcal {G}}_{C-C_0, \frac{1}{2}}$ as $t\rightarrow \infty$.

On the other hand, for every $C<C_0/2$ and every $s \in {\mathbb {R}}$, there exists $\omega _0 \in {\mathcal {G}}_{C, \frac{1}{2}}$ such that $\sup _{t \in [0,\infty )} \Vert \omega (t)\Vert _{H^{\sigma }}=\infty$ for any $\sigma \geqq s$ but such that $\omega (t)$ converges in $H^{s-}$ as $t\rightarrow \infty$.

Furthermore, for $s\geqq 0$ the corresponding velocity field converges strongly in $L^2$ to a shear flow as $t\rightarrow \infty$. Linear inviscid damping holds despite the divergence of $\omega (t)$ in higher regularity.

#### Proof

We proceed similarly as in the case of Theorem 6. Consider a frequency $\eta \gg 1$ and let $1\ll k \leqq \sqrt{\eta }$ to be fixed later. Then by the local well-posedness established in Section 2 we may prescribe smooth initial data $\omega _0^{\eta ,k}$ such that at the time $t=\frac{\eta }{k}$, $\omega$ is given by $e^{i\eta y + ik x}$. Then, we iteratively apply Theorem 8 to obtain that after time $t=2\eta$, the mode $e^{i \eta y + i x}$ is the largest (within a factor) and of size

$$\begin{aligned} C^{k} \left( \frac{\eta ^{k}}{(k!)^2} \right) ^{\gamma }, \end{aligned}$$

where we used that $\xi =\frac{\eta }{k^2}$ changes in each step. We may choose $k=k_\eta$ to maximize this product, which leads to factor $\exp ({\tilde{C}}\gamma \sqrt{\eta })=:g(\eta )$. Furthermore, by Proposition 1 after this time $t=2\eta$, the evolution is asymptotically stable and a small perturbation of the identity.

Let now $\psi \in H^{s}({\mathbb {R}})$ be given and consider the initial datum:

$$\begin{aligned} \omega _0= \int _{\eta } \frac{1}{g(\eta )} {\tilde{\psi }}(\eta ) \omega _0^{\eta ,k_\eta }. \end{aligned}$$

Then by the definition of $g(\eta )$ and the properties of the evolution, $\omega (t)$ will asymptotically to leading order be given by

$$\begin{aligned} \omega _\infty = e^{ix}\int _{\eta } {\tilde{\psi }}(\eta ) e^{i \eta y} \,\mathrm{d}\eta = e^{ix} \psi (y). \end{aligned}$$

We can thus prescribe final data. We further observe that $\frac{1}{g(\eta )}\leqq \exp (-C \sqrt{\eta })$ and thus $\omega _0 \in {\mathcal {G}}_{C,\frac{1}{2}}$. Our proof thus concludes by choosing $\psi \in H^{s}\setminus H^{\sigma }$ appropriately. $\quad \square$

## Discussion

In view of applications to the nonlinear dynamics we note that we have several competing (de)stabilizing effects, whose interaction makes this a very challenging problem:

- On the one hand the norm inflation mechanism of Section 5 and similarly discussed in [5] shows that the vorticity may exhibit instability unless it is initially small in a sufficiently strong Gevrey class.
- On the other hand resonant times are well-separated and for any given $\eta$ there are no resonances after time $\eta$. In particular, in the present problem fixing any finite radius *R* as a frequency cut-off ${\mathcal {F}}^{-1}\chi _{B_R}{\mathcal {F}} \omega$ and its corresponding velocity field do converge irrespective of the regularity of the initial data.
- Any instability will thus have to sustain a sequence of infinitely many separate echo chains for a sequence of times tending to infinity to ensure that the flow is not asymptotically stable after all (see Sections 2 and 5.3).
- While such a sequence of echo chains can be constructed in our model due to its decoupling structure (see Section 5.3), in the full nonlinear problem the conservation of enstrophy limits the possible relative growth. That is the conservation law imposes a hard ceiling for instability in that the $L^2$ energy remains bounded uniformly. Hence, it might be that the linear(!) instability mechanism of echoes is only applicable for finite times, after which the enstrophy limits further growth and the asymptotic stability of Section 2 takes over. Here the modified scattering results of Section 5.3 and [16] provide a first indication that this may result in non-trivial but asymptotically stable behavior.

We further stress that, while stability of the linearized problem in Sobolev [12, 14, 15] and Gevrey spaces [8] is fundamental to attack the nonlinear problem, this article shows that it is further essential to understand the linearization around non-shear low frequency perturbations, which appear naturally in the nonlinear problem.

In the present work we have, for simplicity of calculation and presentation, considered a single-mode perturbation $c\cos (x)$. We expect analogous results to also hold for more general finite sums of small frequency perturbations, though involving quite involved calculations. Our choice $\cos (x)$ is motivated by its simplicity and the fact that, as an eigenfunction of the Laplacian, it is a stationary solution of the Euler equations in Lagrangian coordinates with respect to Couette flow.

## Special Functions and a Proof of Theorem 4

As mentioned in Section 4, the problem

$$\begin{aligned} \partial _t^2 u + \frac{c^2}{\xi ^{-2}+t^2} u=0, t \in (-1,1) \end{aligned}$$

allows for an explicit solution in terms of special functions, which we discuss in the following. Since these explicit solutions are not available for perturbed coefficients or stable under further perturbation, in Sections 4 and 5 we have instead opted to study (and control) the evolution on three intervals $I_1, I_2, I_3$ and, in particular, establish the growth and decay of *u* and $\partial _tu$ on each interval. The interaction of these growths and decays then results in the same power law growth as we observe in the following for the special functions:

### Proof of Theorem 4

Denoting $\xi =\frac{k^2}{\eta }$ for simplicity of notation, one may obtain the following explicit solution for boundary conditions $u(-1)=1, \partial _tu(-1)=0$ (e.g. using Mathematica)

68 $$\begin{aligned}&\big ( -3 c^2 t \, _2F_1\left( \frac{3}{4}-\frac{1}{4} \sqrt{1-4 c^2},\frac{1}{4} \sqrt{1-4 c^2}+\frac{3}{4};\frac{3}{2};-\frac{1}{\xi ^2}\right) \nonumber \\&\quad _2F_1\left( \frac{1}{4}-\frac{1}{4} \sqrt{1-4 c^2},\frac{1}{4} \sqrt{1-4 c^2}+\frac{1}{4};\frac{3}{2};-\frac{t^2}{\xi ^2}\right) \nonumber \\&\quad -c^2 \, _2F_1\left( \frac{5}{4}-\frac{1}{4} \sqrt{1-4 c^2},\frac{1}{4} \sqrt{1-4 c^2}+\frac{5}{4};\frac{5}{2};-\frac{1}{\xi ^2}\right) \nonumber \\&\quad _2F_1\left( -\frac{1}{4} \sqrt{1-4 c^2}-\frac{1}{4},\frac{1}{4} \sqrt{1-4 c^2}-\frac{1}{4};\frac{1}{2};-\frac{t^2}{\xi ^2}\right) \nonumber \\&\quad +3 \xi ^2 \, _2F_1\left( \frac{1}{4}-\frac{1}{4} \sqrt{1-4 c^2},\frac{1}{4} \sqrt{1-4 c^2}+\frac{1}{4};\frac{3}{2};-\frac{1}{\xi ^2}\right) \nonumber \\&\quad _2F_1\left( -\frac{1}{4} \sqrt{1-4 c^2}-\frac{1}{4},\frac{1}{4} \sqrt{1-4 c^2}-\frac{1}{4};\frac{1}{2};-\frac{t^2}{\xi ^2}\right) \big )\nonumber \\&\quad / \big (3 c^2 \, _2F_1\left( \frac{1}{4}-\frac{1}{4} \sqrt{1-4 c^2},\frac{1}{4} \sqrt{1-4 c^2}+\frac{1}{4};\frac{3}{2};-\frac{1}{\xi ^2}\right) \nonumber \\&\quad _2F_1\left( \frac{3}{4}-\frac{1}{4} \sqrt{1-4 c^2},\frac{1}{4} \sqrt{1-4 c^2}+\frac{3}{4};\frac{3}{2};-\frac{1}{\xi ^2}\right) \nonumber \\&\quad -c^2 \, _2F_1\left( -\frac{1}{4} \sqrt{1-4 c^2}-\frac{1}{4},\frac{1}{4} \sqrt{1-4 c^2}-\frac{1}{4};\frac{1}{2};-\frac{1}{\xi ^2}\right) \nonumber \\&\quad _2F_1\left( \frac{5}{4}-\frac{1}{4} \sqrt{1-4 c^2},\frac{1}{4} \sqrt{1-4 c^2}+\frac{5}{4};\frac{5}{2};-\frac{1}{\xi ^2}\right) \nonumber \\&\quad +3 \xi ^2 \, _2F_1\left( -\frac{1}{4} \sqrt{1-4 c^2}-\frac{1}{4},\frac{1}{4} \sqrt{1-4 c^2}-\frac{1}{4};\frac{1}{2};-\frac{1}{\xi ^2}\right) \nonumber \\&\quad _2F_1\left( \frac{1}{4}-\frac{1}{4} \sqrt{1-4 c^2},\frac{1}{4} \sqrt{1-4 c^2}+\frac{1}{4};\frac{3}{2};-\frac{1}{\xi ^2}\right) \big ) \end{aligned}$$

Here $_2F_1$ denotes a hypergeometric function. We may then evaluate this formula at $t=1$ and use the series expansion of $_2 F_1(a,b,c,x)$ at $x=\infty$:

69 $$\begin{aligned}&x^{-a-b} \Bigg (x^b \left( \frac{(-1)^{-a} \varGamma (b-a) \varGamma (c)}{\varGamma (b) \varGamma (c-a)}+\frac{(-1)^{-a} a (a-c+1) \varGamma (b-a) \varGamma (c)}{(a-b+1) \varGamma (b) \varGamma (c-a) x}+O\left( \left( \frac{1}{x}\right) ^2\right) \right) \end{aligned}$$

70 $$\begin{aligned}&\quad +x^a \left( \frac{(-1)^{-b} \varGamma (a-b) \varGamma (c)}{\varGamma (a) \varGamma (c-b)}+\frac{(-1)^{-b} b (b-c+1) \varGamma (a-b) \varGamma (c)}{(-a+b+1) \varGamma (a) \varGamma (c-b) x}+O\left( \left( \frac{1}{x}\right) ^2\right) \right) \Bigg ). \end{aligned}$$

This then, for example, yields that, for $u(-1)=1,u'(-1)=0$,

$$\begin{aligned} u(1)=\,&\big (-3 c^2 \, _2F_1\left( \frac{1}{4}-\frac{1}{4} \sqrt{1-4 c^2},\frac{1}{4} \sqrt{1-4 c^2}+\frac{1}{4};\frac{3}{2};-\frac{1}{r^2}\right) \\&\quad _2F_1\left( \frac{3}{4}-\frac{1}{4} \sqrt{1-4 c^2},\frac{1}{4} \sqrt{1-4 c^2}+\frac{3}{4};\frac{3}{2};-\frac{1}{r^2}\right) \\&-c^2 \, _2F_1\left( -\frac{1}{4} \sqrt{1-4 c^2}-\frac{1}{4},\frac{1}{4} \sqrt{1-4 c^2}-\frac{1}{4};\frac{1}{2};-\frac{1}{r^2}\right) \\&_2F_1\left( \frac{5}{4}-\frac{1}{4} \sqrt{1-4 c^2},\frac{1}{4} \sqrt{1-4 c^2}+\frac{5}{4};\frac{5}{2};-\frac{1}{r^2}\right) \\&+3 r^2 \, _2F_1\left( -\frac{1}{4} \sqrt{1-4 c^2}-\frac{1}{4},\frac{1}{4} \sqrt{1-4 c^2}-\frac{1}{4};\frac{1}{2};-\frac{1}{r^2}\right) \\&_2F_1\left( \frac{1}{4}-\frac{1}{4} \sqrt{1-4 c^2},\frac{1}{4} \sqrt{1-4 c^2}+\frac{1}{4};\frac{3}{2};-\frac{1}{r^2}\right) \big )\\&/ \big (3 c^2 \, _2F_1\left( \frac{1}{4}-\frac{1}{4} \sqrt{1-4 c^2},\frac{1}{4} \sqrt{1-4 c^2}+\frac{1}{4};\frac{3}{2};-\frac{1}{r^2}\right) \\&_2F_1\left( \frac{3}{4}-\frac{1}{4} \sqrt{1-4 c^2},\frac{1}{4} \sqrt{1-4 c^2}+\frac{3}{4};\frac{3}{2};-\frac{1}{r^2}\right) \\&-c^2 \, _2F_1\left( -\frac{1}{4} \sqrt{1-4 c^2}-\frac{1}{4},\frac{1}{4} \sqrt{1-4 c^2}-\frac{1}{4};\frac{1}{2};-\frac{1}{r^2}\right) \\&_2F_1\left( \frac{5}{4}-\frac{1}{4} \sqrt{1-4 c^2},\frac{1}{4} \sqrt{1-4 c^2}+\frac{5}{4};\frac{5}{2};-\frac{1}{r^2}\right) \\&+3 r^2 \, _2F_1\left( -\frac{1}{4} \sqrt{1-4 c^2}-\frac{1}{4},\frac{1}{4} \sqrt{1-4 c^2}-\frac{1}{4};\frac{1}{2};-\frac{1}{r^2}\right) \\&_2F_1\left( \frac{1}{4}-\frac{1}{4} \sqrt{1-4 c^2},\frac{1}{4} \sqrt{1-4 c^2}+\frac{1}{4};\frac{3}{2};-\frac{1}{r^2}\right) \big ) \end{aligned}$$

can be approximated as

$$\begin{aligned} \frac{r^{-\sqrt{1-4 c^2}} r^2 \left( -\frac{3 \left( 2^{\sqrt{1-4 c^2}-2} c^2 \left( \sqrt{1-4 c^2}+1\right) \varGamma \left( \frac{1}{2} \sqrt{1-4 c^2}\right) ^2\right) }{\varGamma \left( \frac{1}{2} \left( \sqrt{1-4 c^2}+3\right) \right) ^2}\right) +o}{3 r^2+o}, \end{aligned}$$

where we denoted $r=\xi ^{-1}\ll 1$ and *o* refers to terms decaying to higher order in *r*. We note in particular that the powers $r^2$ cancel. Approximating the value of the $\varGamma$ functions by their value in $c=0$, we thus obtain

$$\begin{aligned} \xi ^{\gamma } \pi c^2. \end{aligned}$$

Similar calculations for $\partial _tu$ and other initial data lead to the following coefficient matrix:

$$\begin{aligned} \begin{pmatrix} u(1)\\ \partial _tu(1) \end{pmatrix}\approx \xi ^\gamma \begin{pmatrix} \pi c^2 &{} -\pi c^2 \\ \pi c^2 &{} -\pi c^2 \end{pmatrix} \begin{pmatrix} u(-1)\\ \partial _tu(-1) \end{pmatrix} \end{aligned}$$

$$\square $$

While the above calculations and asymptotics are explicit, they are also rather opaque. The splitting of the evolution into intervals $I_1,I_2,I_3$ studied in Lemmas 3,  4 and Proposition 3 instead provides a much clearer view of the underlying mechanism and yields the same leading asymptotics in terms of $\xi$.
